# A machine learning-based data mining in medical examination data: a biological features-based biological age prediction model

**DOI:** 10.1186/s12859-022-04966-7

**Published:** 2022-10-03

**Authors:** Qing Yang, Sunan Gao, Junfen Lin, Ke Lyu, Zexu Wu, Yuhao Chen, Yinwei Qiu, Yanrong Zhao, Wei Wang, Tianxiang Lin, Huiyun Pan, Ming Chen

**Affiliations:** 1 Zhejiang Provincial Center for Disease Control and Prevention, Hangzhou, 310051 China; 2 College of Biosystems Engineering and Food Science, Zhejiang University, Hangzhou, 310058 China; 3 College of Life Sciences, Zhejiang University, Hangzhou, 310058 China; 4 The First Affiliated Hospital of School of Medicine, Zhejiang University, Hangzhou, 310058 China

**Keywords:** Biological age, Biological features, Machine learning, Interpolation, Stacking, Health status

## Abstract

**Background:**

Biological age (BA) has been recognized as a more accurate indicator of aging than chronological age (CA). However, the current limitations include: insufficient attention to the incompleteness of medical data for constructing BA; Lack of machine learning-based BA (ML-BA) on the Chinese population; Neglect of the influence of model overfitting degree on the stability of the association results.

**Methods and results:**

Based on the medical examination data of the Chinese population (45–90 years), we first evaluated the most suitable missing interpolation method, then constructed 14 ML-BAs based on biomarkers, and finally explored the associations between ML-BAs and health statuses (healthy risk indicators and disease). We found that round-robin linear regression interpolation performed best, while AutoEncoder showed the highest interpolation stability. We further illustrated the potential overfitting problem in ML-BAs, which affected the stability of ML-Bas’ associations with health statuses. We then proposed a composite ML-BA based on the Stacking method with a simple meta-model (STK-BA), which overcame the overfitting problem, and associated more strongly with CA (r = 0.66, *P* < 0.001), healthy risk indicators, disease counts, and six types of disease.

**Conclusion:**

We provided an improved aging measurement method for middle-aged and elderly groups in China, which can more stably capture aging characteristics other than CA, supporting the emerging application potential of machine learning in aging research.

**Supplementary Information:**

The online version contains supplementary material available at 10.1186/s12859-022-04966-7.

## Introduction

In the context of global aging, exploring the representation methods, evaluation indicators, and influencing factors of aging based on big medical data has become an important social issue and a new research hotspot [1]. Aging is an organismal phenomenon manifested by an increased chance of healthy risk (e.g. the likelihood of disease, death) or decreased function over time [2]. The introduction of biological age (BA) is a critical step in aging research. BA is an ideal indicator to provide evidence on aging independent of chronological age (CA) and measures the rate of human aging associated with the functional decline more accurately than CA [3, 4]. Besides, BA is closely related to health characteristics such as physical function, cognition, morbidity, and mortality by measuring the cumulative level of impairment [5]. Effective BA construction methods and quantitative assessments of the associations between BA with health status will contribute to further understanding of aging and provide effective risk stratification [6, 7].

Current BAs are mainly based on statistical models of a series of biological features [8]. These features include clinical indicators [4, 9, 10], instrumental parameters [11, 12], and molecular genetic measures [13, 14]. The methods commonly used in BA models are based on univariate or multivariate regression methods [7], such as principle component analysis (PCA) [15], multilayer perceptron (MLP) [16], and the Klemera and Doubal method (KDM) [17]. Although these classical methods perform well in predicting adverse aging outcomes, they have limitations in processing multidimensional data, especially when the shape of the distribution is not suited for parametric methods [18], and recognizing the actual interactions between the biomarkers and outcomes [19], as some significant biomarkers were proved to be nonlinear [17]. While recently, new approaches applying machine learning (ML) algorithms have shown considerable accuracy and efficiency in BA prediction [20, 21], causing wide attention [22]. Furthermore, the stacking and bagging algorithm displays better performance in distinguishing significant features [23], revealing the complicated non-linear relationships between biomarkers and the target condition [24], but few applications in ML-BA construction.

The Pearson correlations, MAE, and RMSE between BA and CA are the preferred and most commonly used indicators to compare different BA estimation algorithms [25, 26]. Exploring the associations of ML-BA with epidemiological variables (e.g. health risk indicators, mortality), genetic and environmental factors, and common age-related chronic diseases (e.g. heart disease, kidney disease) can further examine its potential as a biomarker of aging in the general population [6, 27, 28]. Notably, we found in the previous ML-BAs that the correlations between BA and CA attained from the test data used for comparing model performances, and the full data, including both the training data and test data, showed obvious differences. [18, 29]. The reason might be that overfitting makes the model outperform the test set on the training set. And then the model trained on the training set predicted the full dataset’s BA, resulting in a different but better BA performance than the test set. Under such circumstances, whether the degree of overfitting will affect the stability of the association results needs to be further considered.

Valid BA and reliable conclusions are usually based on large population data, but complete large datasets for data mining in public health research are rare, as related medical databases are often lost for various reasons [30, 31], such as sample missing [32], human error [33]. A normal method to solve the problem is ignoring samples with missing values. However, omitting the missing data will greatly limit the downstream analysis performance [18]. Hence, using interpolation methods to estimate incomplete datasets, which will contribute to improving the performance of subsequent analysis [34, 35], becomes a more suitable choice. Some machine learning-based (ML-based) methods have exhibited great application potential in recent years [36–39]. However, most of the current studies on BA used relatively complete datasets, or deal with missing values only with the most common methods (filled with mean, median, mode, zero or random values) [18]. Insufficient attention has been paid to the complexity and incompleteness of medical data. Therefore, exploring novel and effective interpolation methods will be a constructive and worthy practice in the data preprocessing before building BA models with physical examination data. Besides, to reduce the influence of overfitting on the results, cross-validation methods should be adopted, such as K-fold cross-validation [40], and generalized cross-validation (GCV) [41].

Additionally, most of the current ML-BA studies were from European and American populations [42, 43], and ML-BA based on large Chinese population data (more than 30,000 people) was still very limited [18]. The correlation of ML-BA with CA will vary due to differences in populations and biomarkers [44]. Constructing ML-BA with a large Chinese population from different sources and linking ML-BA with important health statuses will help to further explore the validity and application potential of ML-BA in the Chinese population.

**Fig. 1:**
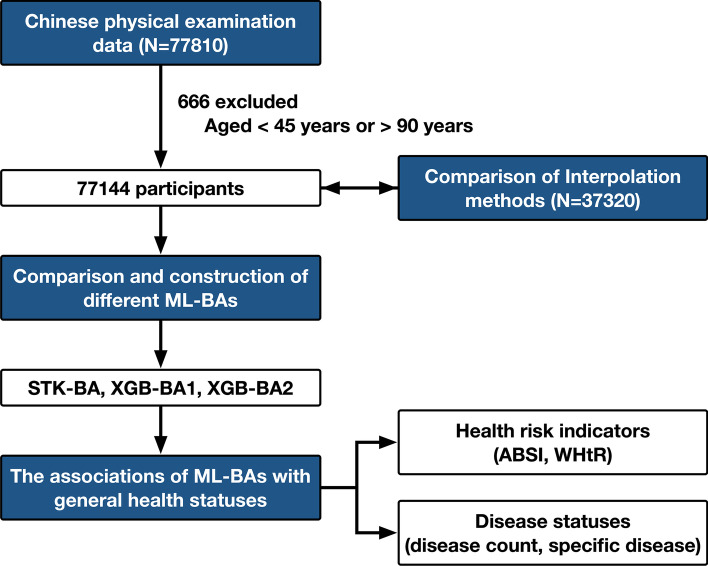
The analytical flowchart of our study. *ML-BA, machine learning-based biological age; STK-BA, staking model-based biological age; XGB-BA, XGBoost-based biological age; ABSI, A Body Shape Index; WHtR, Waist-to-height ratio

In the research, we used medical examination data (45–90 years) in Zhejiang Province, China, and Fig. 1 illustrated our analysis flow. We focused on four aspects: (1) comparing the applicability of different interpolation methods in medical examination data (e.g. round-robin linear regression, AutoEncoder); (2) constructing ML-BAs based on Chinese large population samples with several machine-learning algorithms; (3) examining associations of ML-BAs with health statuses (e.g. health risk indicators, disease status); and most importantly, (4) exploring the influence of overfitting degree on the stability of the associated results and proposed the optimized ML-BA model.

## Results

### Comparison of missing value interpolation methods

**Fig. 2:**
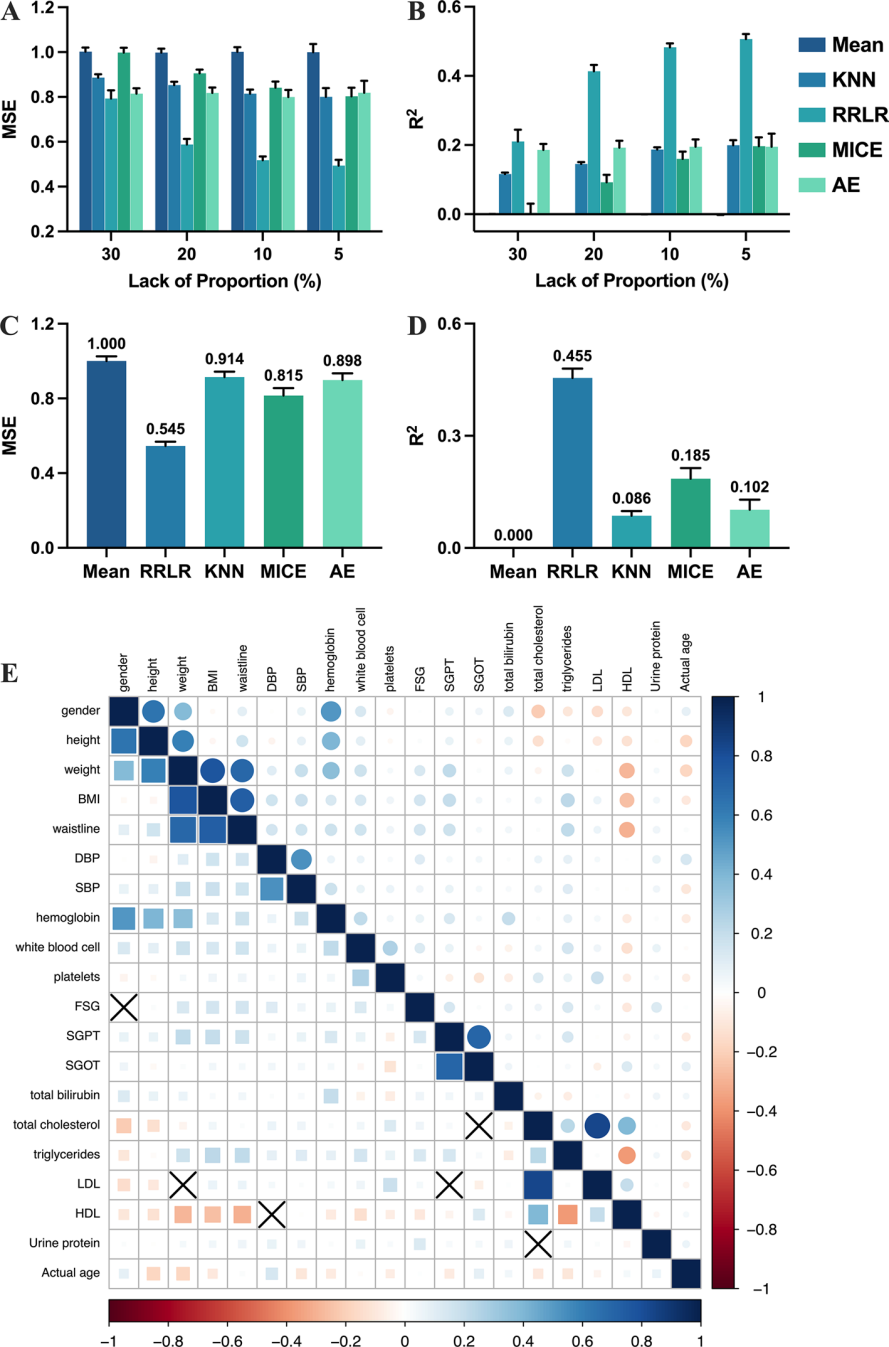
Imputing results of different methods in missing completely at random (MCAR, **A**, **B**) and missing not at random (MNAR, **C**, **D**) simulation datasets. Correlation between biological features and chronological age (**E**)

As shown in Fig. 2A–D, the interpolation results of mean, KNN, AE, RRLR, and MICE for continuous variables on MNAR and MCAR simulation data sets were presented. MSE and R^2^ compared the accuracy and validity of interpolation respectively. The parameter selection process of KNN and MICE was presented in Additional file 1: Table S1 (MCAR) and Additional file 1: Table S2 (MNAR), and the optimal parameter of both models varied with missing proportions (Additional file 1: Tables S1 and S2). Subsequently, the best results were selected and compared with other models under different missing conditions. AE hyper-parameters considered encoder layers, epochs, activation function, batch size, and learning rate. The optimized parameters of AE and RRLR were presented in Additional file 1: Table S3. Additional file 1: Table S4 recorded the interpolation time consumed by the different models.

The results showed that RRLR outperformed other methods under MCAR and MNAR (Fig. 2A–D). The MSE of MICE and RRLR increased significantly with the increase in missing ratio (Fig. 2A, B), but AE showed more excellent stability, with the missing ratio growing from 5 to 30%, and the R^2^ only decreased by 4.61%. The lower the missing rate, the greater the advantage of RRLR, while AE was more suitable for cases with a high missing rate. The results in the MNAR simulation dataset (Fig. 2C, D) were similar to those in MCAR (Fig. 2A, B). RRLR reduces MSE by 33.12% compared to MICE, the second-most accurate interpolation method in MNAR. R^2^ possessed the same trend as MSE, and RRLR interpolation results displayed the best correlation. In addition to interpolation performance, the time spent in interpolation should also be considered (Additional file 1: Table S4). RRLR exhibited a similar time cost to AE and mean, while the time consumed by KNN mainly depended on the missing ratio. MICE needed the most time to complete interpolation. In general, RRLR was used to fill missing values, and the predicted value of the binary variable greater than 0.5 was marked as 1, otherwise, it was 0. BA will be predicted on the new dataset.

### Features selection and BA predictor construction

A total of 22 potential biological features were considered for this study. Additional file 1: Fig. S1 showed the optimized lambda and feature selection process in Lasso regression. Urine sugar, urine occult blood, and urine acetone bodies were excluded (Additional file 1: Table S5 and Fig. S1). Figure 2E presented the correlation between variables, with an ‘X’ mark indicating no significant correlation (P > 0.05). Notably, all features were significant (P < 0.05). These two steps yielded 19 features for estimating BA.

Among the machine learning and neural network models explored, ML-BA predicted by Xgboost showed the highest correlation with CA (Pearson’s r = 0.64 in the test set), while Catboost, LGBM, GBDT, and Extra Tress showed similar results (Table 1). Among the five models, R^2^ ranged from 0.32 to 0.41, and RMSE ranged from 4.49 to 4.89. The parameters of all the above models were detailed in Additional file 1: Table S6. However, the evaluation metrics of these five models were significantly different in training and test set (Table 1), which was attributed to the choice of parameters in the model that greatly affected the model’s fit during training. If over-fitting on the training set was ignored and the model obtained from the training set was used to predict BA of the entire dataset, overfitting will be introduced into the final result, resulting in higher instability of BA. Thus, in addition to determining the optimal model by test set results, the introduction of the prediction results of the overfitting should be avoided in the final prediction.

**Table 1:** RSME, R^2^, MAE, and Pearson’s correlation of ML-BA models

Model	Training set (80%)				Test set (20%)			
	RMSE	R^2^	MAE	Pearson’s correlation	RMSE	R^2^	MAE	Pearson’s correlation
Stacking (SVM)	5.765	0.438	4.349	0.661	5.776	0.435	4.352	0.659
**Stacking (GAM)**	**5.777**	**0.434**	**4.409**	**0.658**	**5.774**	**0.433**	**4.403**	**0.658**
Stacking (MLR)	5.788	0.431	4.418	0.657	5.786	0.431	4.414	0.656
Stacking (RF)	2.786	0.900	2.094	0.949	5.828	0.422	4.444	0.650
XGBoost	4.988	0.578	3.780	0.760	5.869	0.414	4.489	0.643
CatBoost	3.674	0.771	2.739	0.878	5.893	0.409	4.494	0.640
LGBM	4.128	0.711	3.097	0.843	5.926	0.403	4.538	0.634
GBDT	5.513	0.484	4.239	0.696	5.951	0.397	4.579	0.630
Extra Trees	0.000	1.000	0.000	1.000	6.319	0.321	4.889	0.566
DNN	6.251	0.341	4.869	0.584	6.419	0.299	5.014	0.547
CNN	5.918	0.409	4.583	0.640	6.467	0.289	5.016	0.537
GAM	6.516	0.279	5.094	0.529	6.509	0.280	5.072	0.529
MLR	6.692	0.240	5.238	0.490	6.691	0.239	5.224	0.489
AdaBoost	6.986	0.172	5.499	0.414	6.994	0.168	5.501	0.409

Bold indicates the performance of the final selected model

**Fig. 3:**
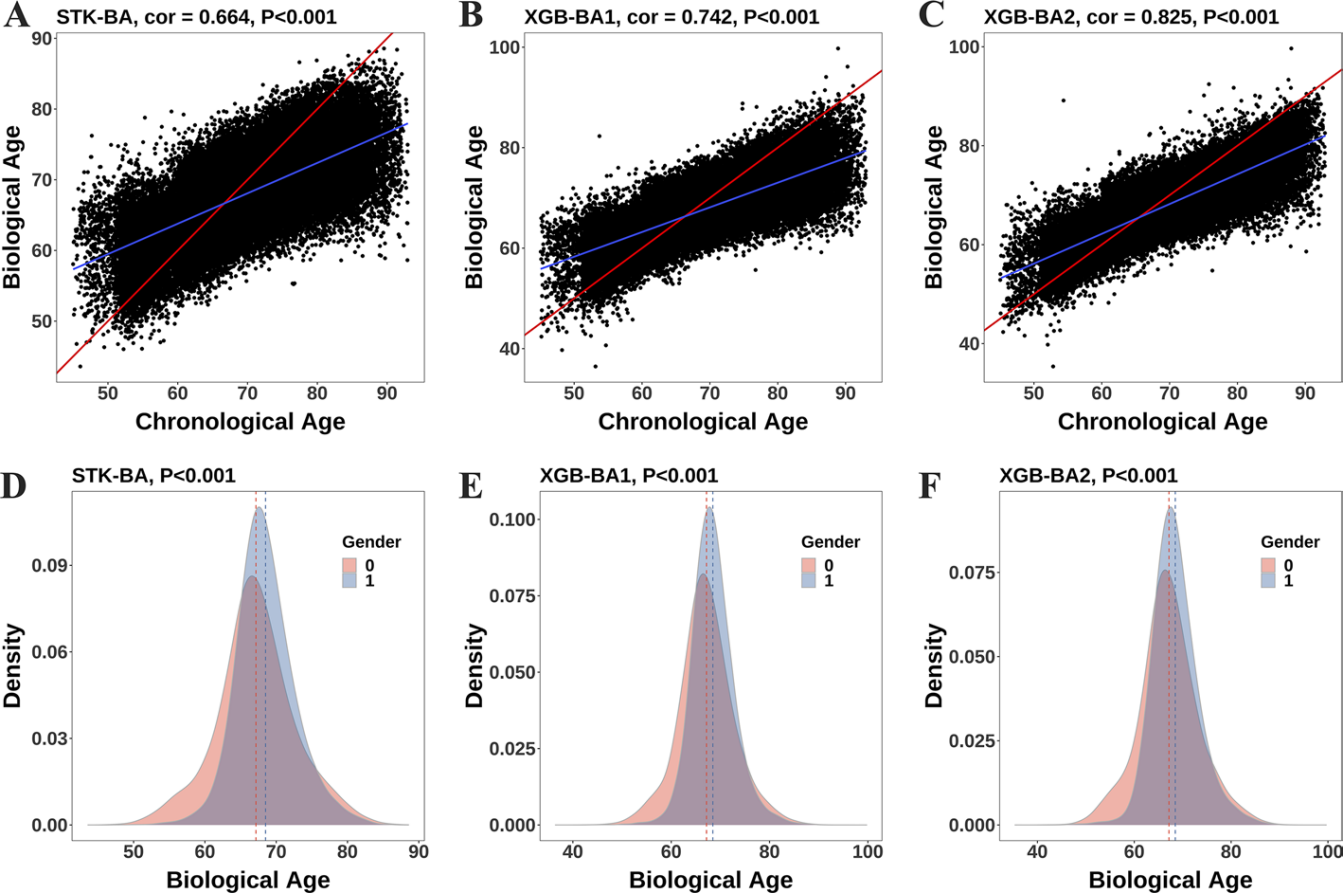
Correlation **A**–**C** between chronological age (CA) and biological age (BA) and distribution **D**–**F** of BA in the whole sample

To this end, we applied the Stacking approach to fusing the model, where the parameters were inherited from a single model. This method could further improve the prediction accuracy besides effectively lowering the interference of the overfitting. Considering the training time, complexity, and fitting effects of the meta-model, the GAM (spline regression) was finally selected to fuse the above five models. The RMSE in the training and test sets were 5.78 and 5.77, respectively, and the R^2^ was both 0.43. Therefore, we used the fusion model with 19 biological characteristics to get STK-BA. The STK-BA of the entire study population ranged from 44 to 89 years (Table 2), with a mean of 67.8 (SD = 5.0). For females, BA ranged from 43 to 88 years, with a mean of 67.2 (SD = 5.6). For males, BA ranged from 47 to 89 years, with a mean of 68.5 (SD = 4.2). Compared with males, BA in the female population was significantly younger (P < 0.001) and tended to be more normally distributed (Fig. 3A). Table 2 presented that STK-BA was significantly correlated with CA (R = 0.660–0.668, P < 0.001).

**Table 2:** Distribution of BA in male and female study populations

BA		Min	Max	Median	Mean (SD)	Correlation with CA (P value)
STK-BA	Male	47.23	88.57	68.17	68.51 (4.16)	0.604–0.617 (< 0.001)
	Female	43.59	88.39	67.02	67.16 (5.58)	0.682–0.692 (< 0.001)
	Total	43.59	88.57	67.61	67.77 (5.03)	0.660–0.668 (< 0.001)
XGB-BA1	Male	43.48	90.94	68.17	68.47 (4.39)	0.695–0.706 (< 0.001)
	Female	36.45	99.75	66.99	67.18 (5.68)	0.756–0.764 (< 0.001)
	Total	36.45	99.75	67.60	67.76 (5.16)	0.738 ~ 0.745 (< 0.001)
XGB-BA2	Male	44.39	92.43	68.08	68.48 (4.82)	0.791–0.799 (< 0.001)
	Female	35.37	99.66	66.96	67.17 (6.23)	0.836–0.842 (< 0.001)
	Total	35.37	99.66	67.54	67.76 (5.67)	0.822–0.827 (< 0.001)

To further highlight the advantages of STK-BA and the influences of over-fitting, we constructed two XGB-BAs with similar performance in the test set (the results and parameters were shown in Additional file 1: Table S7). Although XGB-BA2 and XGB-BA1 had similar results on the test set (0.4% MAE difference), XGB-BA2 further improved the fit of the training set, showing a higher correlation with CA (13.1%-increase). Therefore, as shown in Table 2 and Fig. 3, compared with STK-BA, XGB-BAs showed poorer results in the test set, but both improved the correlation with CA in the whole sample (XGB-BA1: 0.738–0.745; XGB-BA2: 0.822–0.827)., the effect of gender on XGB-BAs was similar to that of STK-BAs, but XGB-BAs exhibited a wider BA range (Table 2 and Fig. 3). Taking XGB-BA2 as an example, compared with STK-BA, the BA range was expanded by 42.9%.

### The importance of features for the stacking model

Additional file 1: Table S8 recorded the feature importance values of the sub-models in the Stacking model, and Additional file 1: Fig. S2 showed the average feature importance value for the Stacking model. DBP, height, SBP, gender, and platelet content were the top 5 biometric characteristics in the Stacking model. Furthermore, weight, SGPT, waist, and SGOT also showed above-average importance. Conversely, the presence or absence of urinary protein was the least essential marker.

### The associations between health risk indicators and STK-BA, XGB-BAs

**Fig. 4:**
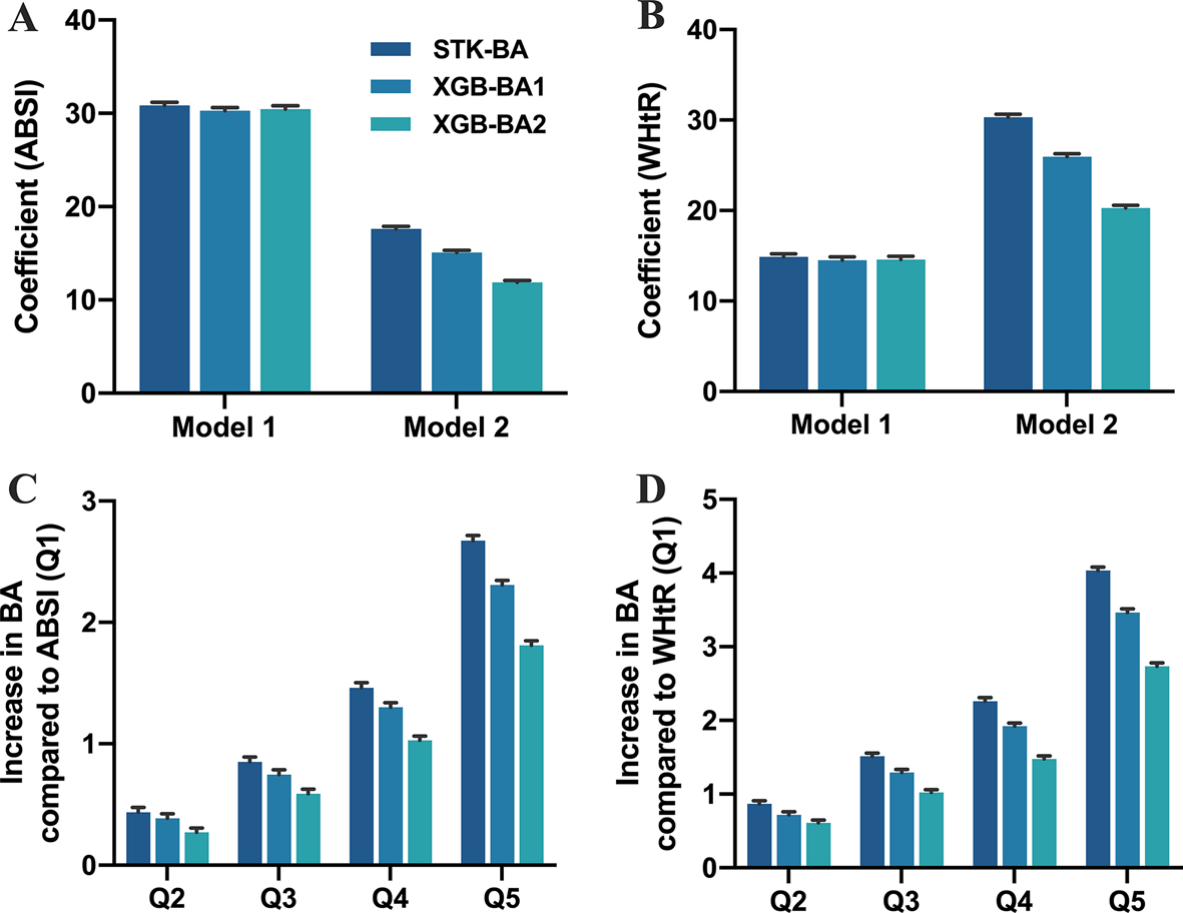
Associations of STK-BA and XGB-BAs with health risk indicators (A Body Shape Index (ABSI), Waist-to-height ratio (WHtR)). Health risk indicators as continuous variables (ABSI: **A**, WHtR: **B**). Health risk indicators as categorical variables (Model 2, ABSI: ** C**, WHtR: ** D**). Model 1 was a crude model, Model 2 was adjusted for CA, BMI, and family disease status

In this evaluation, we chose ABSI and WHtR as health risk status indicators. Previous studies have pointed out that WHtR was a better measure of an individual’s health than BMI [6, 45]. ABSI based on physical characteristics appeared to be an indicator of premature death in the general population, predicting mortality risk across age, gender, and weight [46]. The three BAs were of the same type and therefore numerically comparable. As shown in Additional file 1: Tables S9, S10, and S11 and Fig. 4, we observed all three ML-BAs exhibited significant positive correlations between ABSI and WHtR (P < 0.001). Results did not change after adjusting for covariates of CA, BMI, and family disease (P < 0.001). And, the correlation strength increased from the first quantile to the fifth quantile, showing a consistent trend. This suggested that the association between ML-BAs and health risk was stable. However, not all ML-BAs showed consistent trends. In an anthropometrically constructed DNN model, the log-rank test for SBSI and WHtR quartiles found that the X^2^ statistic increased from Q1 to Q2, then decreased from Q2 to Q3, but the overall (Q1–Q4) showed an increasing trend [6]. It was worth noting that from STK-BA to XGB-BA1 and XGB-BA2, the strength and significance of the association of BAs with two health risk indicators continued to decline according to the model coefficients and t-statistics (Fig. 4 and Additional file 1: Tables S8, S9, S10, S11, and S12). Compared with the Q1 group (Model 2) with the lowest ABSI (WHtR) value (Additional file 1: Tables S9, S10, S11, and S12), STK-BA, XGB-BA1, and XGB-BA2 in the Q5 group increased by 2.67 (4.04), 2.31 (3.47), 1.81 (2.73), respectively. Therefore, the increased degree of overfitting of the model reduced the association between BAs and health risk indicators. It could be inferred that when the association strength was small or the degree of overfitting was too high, ML-BA may no longer be correlated with health risk indicators.

### The associations between disease statuses and STK-BA, XGB-BAs

**Fig. 5:**
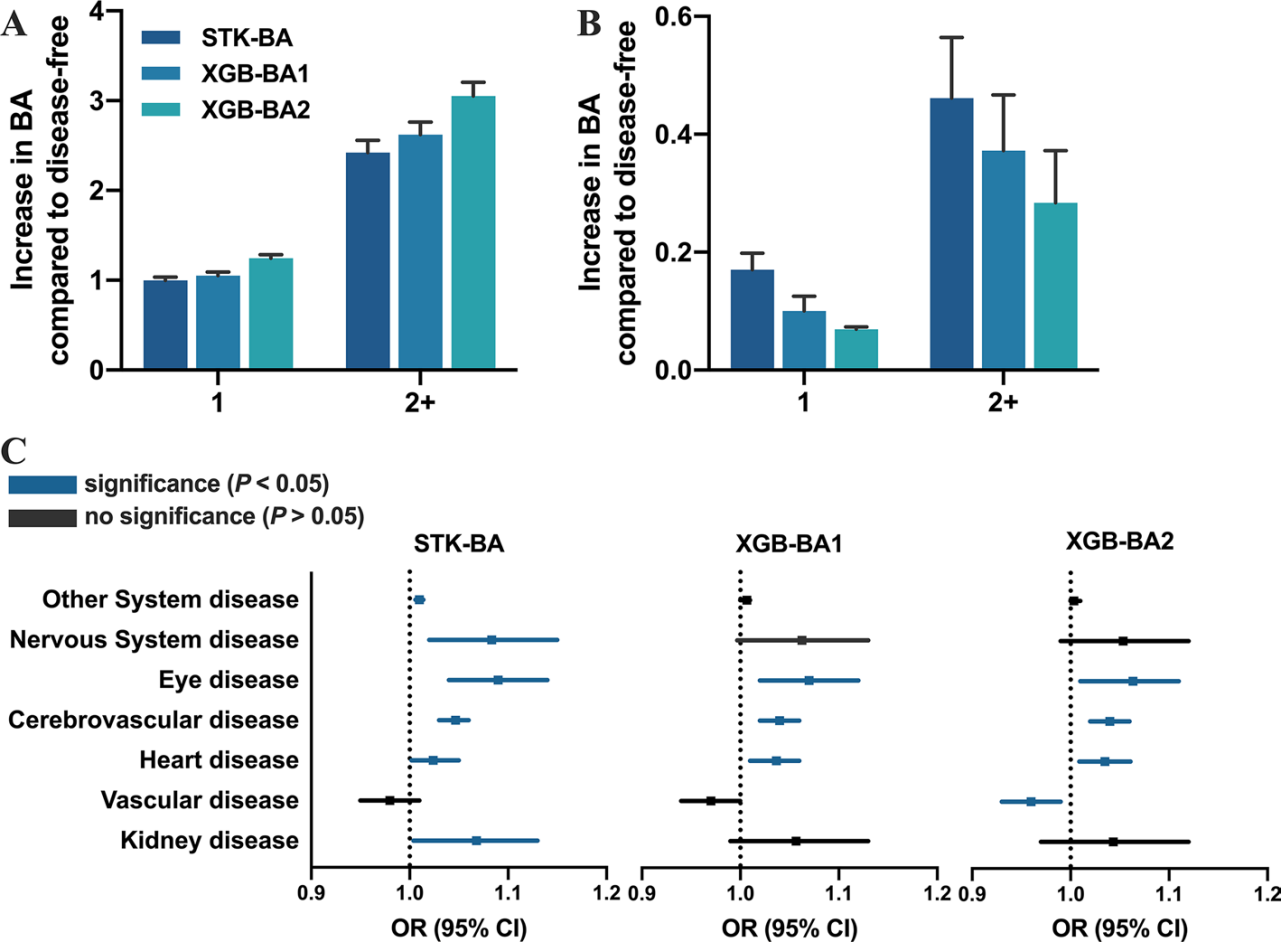
Associations of STK-BA and XGB-BAs with disease counts (**A**: Model 1, **B**: Model 2). The associations between each disease and STK-BA, XGB-BAs (**C**: Model 2). Model 1 was a crude model, Model 2 was adjusted for CA, and family disease status

The increase in STK-BA and XGB-BAs counted for each disease compared to disease-free participants was shown in Fig. 5A, B, Table 3 and Additional file 1: Table S12. Overall, participants with the disease had higher STK-BA and XGB-BAs, and the results remained significant after adjusting for CA and family disease (P < 0.01). In Model 1, XGB-BA2 had the largest BA response to disease count change, while STK-BA had the smallest. Compared with those without the disease, for STK-BA, XGB-BA, and XGB-BA2, those with 1 disease were 0.998, 1.053, and 1.240 years older, and those with 2 or more diseases were 2.422, 2.623, and 3.047 years older. Interestingly, after adjusting for covariates (CA and family disease status), the results were just the opposite. Those with 1 disease were 0.170, 0.100, and 0.069 years older than those without the disease, and were 0.461, 0.372, and 0.284 years older than those with 2 diseases. Also changing was the significance between disease counts and BA (Model 2), the least significant for XGB-BA2 (1:0.024, 2+ : 0.001) and the strongest for STK-BA (1: < 0.001, 2+ : < 0.001).

**Table 3:** Associations of STK-BA and XGB-BAs with disease counts

	Model 1*			Model 2**		
	Coef (SE)	t-value	P	Coef (SE)	t-value	P
STK-BA	0.025 (0.001)	24.20	< 0.001	0.008 (0.001)	5.981	< 0.001
XGB-BA1	0.025 (0.001)	25.08	< 0.001	0.006 (0.002)	4.130	< 0.001
XGB-BA2	0.023 (0.001)	26.76	< 0.001	0.005 (0.002)	3.205	0.001

*Model 1 was a crude model

**Model 2 was adjusted for CA, and family disease status

Poisson regression models were used to examine the associations between BAs and disease counts in the full sample (Table 3). Both STK-BA and XGB-BAs were significantly associated with disease counts (P < 0.001). Consistent results (P < 0.01) were observed after adjusting for CA and family diseases, although the absolute values decreased. Consistent with the trend in the linear regression model, STK-BA showed the strongest association with disease counts (Model 2: Coef = 0.008, SE = 0.001), while XGB-BA2 was the weakest (Model 2: Coef = 0.005, SE = 0.002).

To gain further insights into the relations between the BAs and disease counts, the associations between each disease and STK-BA, XGB-BAs were explored (Fig. 5C and Additional file 1: Table S13). As expected, STK-BA showed a significant positive correlation (P < 0.05) with almost all diseases (except for vascular disease, P = 0.190). XGB-BA1 showed no significant association with vascular disease, kidney disease, and nervous system disease. Notably, in addition to being unrelated to kidney, eye, and nervous system disease, XGB-BA2 was significantly negatively correlated with vascular disease (OR: 0.96, 95% CI: 0.93–0.99) with vascular disease. Furthermore, it was found from the z-score and P values in Additional file 1: Table S13 that compared with XGB-BA1, the associations between XGB-BA2 and diseases (except vascular diseases) were further weakened. This illustrated that overfitting would lead to obvious instability in the results. This also explained why, after adjusting for CA and family disease, XGB-BAs showed weaker associations with disease counts as overfitting degree increased. However, our proposed STK-BA showed fascinating results. After adjusting for covariates, each 1-year increase in STK-BA was associated with a 7% increase in the risk of developing kidney disease (OR: 1.07, 95% CI: 1.00–1.13), 2% for heart disease (OR: 1.02, 95% CI: 1.00–1.05), 5% for cerebrovascular disease (OR: 1.05, 95% CI: 1.03–1.06), 9% for eye disease (OR: 1.09, 95% CI: 1.04–1.17), 8% for nervous system disease (OR: 1.08, 95% CI: 1.02 ~ 1.15) and 1% for other system diseases (OR: 1.01, 95% CI: 1.01–1.01). The results were similar to previous studies. BA has been attested to be a strong indicator and predictor of multiple morbidities, especially chronic diseases [47, 48]. This might be attributed that diseases are closely related to aging. One study showed a stronger association between BA and all-cause morbidity than CA or the traditional biomarkers of age-related diseases (Hazard ratio 1.06 vs. 1.05 and 1.03), including stroke, dementia, Alzheimer’s disease, cancer, coronary heart disease, and diabetes mellitus [49].

## Discussion

There is no general missing value interpolation method, but only the most appropriate one. We compared five classical but effective methods for the Chinese physical examination data and found that RRLR performed best under the same missing ratio of the original data. However, the superior performance of the RRLR method was not universal, and it was more suitable for low missing ratios (e.g. less than 30%). This is because the strategy of RRLR is to build regression models to predict and impute the missing features according to other complete samples in an iterative loop [50]. Although this strategy allows RRLR to utilize as many observations as possible during interpolation, regression typically requires many samples with non-missing data to produce stable results [33]. Therefore, under the condition of MCAR, the performance of RRLR interpolation results will decrease significantly with the increase of the missing rate. However, since the overall missing rate in MNAR data is only about 5%, the RRLR model is suitable. Likewise, Yu et al. [51] pointed out that multiple regression imputation was suitable for filling in the missing in the WHO ARI Multicentre Study of clinical signs and etiologic agent dataset. In addition, MICE is widely used for interpolation in medical data, but is usually used in cases assuming missing at random (MAR) [52, 53]. Introduction of missing data through MCAR and MNAR may lead to poor MICE performance. Hegde H pointed out that MICE was suitable for situations with fewer missing variables and fewer missing data [53], which explained why the performance of MICE decreased significantly with an increasing missing rate in MCAR, but was second only to RRLR in MNAR. AE interpolation showed the best stability. As a common artificial neural network in deep learning, deep AE can perform representation learning on the input information, form a higher-level feature map, and then reconstruct the data at the output, reducing sensitivity to higher missing rates [54, 55]. Furthermore, AE has the advantage of capturing more complex or nonlinear relationships between inputs and has a highly robust noise reduction capability [34, 56]. An important problem in data interpolation is dimensionality reduction. The large data vector is reduced to a smaller data vector after interpolation, which shows better results in electronic health record data [57]. AE typically includes the coding layers leading to a central part, followed by the symmetric decoding layers. The symmetrical structure and the central part offer an internal representation of the input data with lower dimensions and thus have the advantages described above [34]. Peralta M also found that when AE was trained on 10–40% missing data, the accuracy index did not change significantly [34]. Furthermore, according to the variance of R^2^ and MSE, the results are stable and convincing. However, given the uneven distribution of physical examination data, LOOCV or GCV can be introduced when the results are highly biased [58, 59].

More importantly, we found an interesting phenomenon in the previous Chinese population-based ML-BA, which had not been discussed before. When the correlation or R^2^ between BA and CA was taken as the criterion, the results on the test set were quite different from the final prediction of BA on the full dataset. Taking the previous XGB-BA as an example, the R^2^ of the model in the test set was 0.27, while the correlation between BA and CA was 0.75 in the final results (BA to CA regression belonged to simple linear regression, so R = cor = 0.75, R^2^ = 0.56) [18]. The same was also found in the XGB-BA based on the Dongfeng–Tongji cohort [29]. This might be because the model trained on the training set predicted BA on the full dataset, which introduced interference from parameter tuning and training overfitting. However, this still requires further confirmation, as previous studies did not explicitly state how the model was obtained when it finally predicted BA. In any case, the consistency of the test set with the final results is what we would expect.

The correlation between BA and CA was usually regarded as an indispensable index to evaluate BA prediction models. However, after selecting the best model, how to obtain stable correlation analysis results with BA in the whole sample is also of high value. Two generally used health statuses (health risk indicators and disease status) were used as different evaluation aspects to illustrate the influence of different overfitting degrees on correlation strength and significance in ML-BAs. We found that even with similar test set results, as the overfitting degree increased, XGB-BA2 exhibited less obvious associations with health risk indicators (ABSI, WHtR), disease counts, nervous system disease, and eye disease. This finding suggested that the results of association analysis would vary due to parameter selection and other reasons. This can be attributed to the fact that the core purpose of BA is to capture aging features beyond CA, while overfitting causes the model to over-learn the CA feature of the training set. Cao et al. [38] adopted default parameters in the model to overcome this problem, but it did not work fundamentally.

To avoid overfitting affecting the stability of the association results between BA and health outcomes in the entire dataset, we propose three possible solutions. The first is to let the model show basically the same fitting results on the training set and test set, which is the most convenient and least expensive. Secondly, the method of using cross-modeling to predict, such as LOOCV or K-fold, always keeps the final predicted samples from participating in the construction of the model, but this will produce multiple models that are not exactly the same. The prediction accuracy of each model also usually varies due to parameters and different training samples. Therefore, this method presents new challenges for practical application and less time cost. The third is to use only the sample results on the test set for further analysis, but this does not meet the principle of maximizing the use of data and reduces the reliability of the results.

For this case, our proposed STK-BA could improve the correlation between BA and CA while maintaining the consistency of the model results (the correlation of the training set and the test set are the same in three decimal places). What’s more, the positive association of STK-BA with health risk indicators, disease counts, and specific diseases was also more pronounced, suggesting that it better captures the aging-related features behind diseases. This may be attributed to the biological features we considered to represent different physiological functions or dimensions: immune system (e.g. platelet count, white blood cell), cardio-metabolic system (e.g. HDL, DBP), liver function (e.g. SGPT, SGOT), phenotypic dimension (e.g. height, waist), kidney injury (e.g. urine protein). Additionally, the associations we considered included eye disease and kidney disease, which were also not covered in previous Chinese population studies [26].

The Stacking method we adopted is a mechanism to combine the learned types of models into one, consisting of base models and a meta-model [60]. Instead of selecting a model from multiple models for generalization or simple averaging, Stacking uses a meta-model to balance the features (the output of the base model) and predict [33], which is somewhat like a two-layer neural network. Cross-validation of base models and the simple meta-model are the keys to overcoming the overfitting influences. Because the new training and test sets (as input to the second layer) are derived from the predicted values of data sets other than the ones used to build the model, overfitting during training will not be introduced. Meanwhile, the combined data of prediction values from several different models makes the new data sets cover more potential features, which provides support for better prediction performance. Furthermore, a simple meta-model, such as linear regression and generalized additive model, can well reduce the possibility of model overfitting and have good generalization ability, so that the meta-model has similar fitting effects in the training set and test set in the second layer. However, an overly complex meta-model will also lead to overfitting. We observed this when utilizing RF as a meta-model (Table 1). Overall, while outperforming the single base model, Stacking model can overcome the difficulties of overfitting and obtain stable predicted BA on the whole sample for association analysis. More importantly, the Stacking method is equally applicable to the BA based on a single model and can be further generalized.

The correlation between our STK-BA and CA (r = 0.66) on the test set was better than previously published BA (r = 0.52) based on 19 blood biomarkers [18] but weaker than BA (r = 0.74) which considered 44 biomarkers including lung function. This phenomenon is plausible, depending on the population-specific and age-related biosignatures in different datasets [29]. However, it is worth noting that we showed better CA correlations with the same number of biomarkers in the Chinese population. Additionally, Mamoshina et al. [44] found that models trained in a given population declined in correlation when tested across ethnicities (given population: R^2^ ranged from 0.49 to 0.69; different populations: R^2^ ranged from 0.24 to 0.34). ML-BA would exhibit different correlations with CA due to differences in population and biometrics [44] Therefore, we constructed ML-BA using Chinese populations from different sources, and this helped to further confirm the applicability of ML-BA in the Chinese population by associating aging measures with important health conditions and outcomes.

DBP, height, SBP, gender, and platelet content were the five most essential variables screened out in the Stacking model, which may play a vital role in assessing BA differences in different populations. In fact, DBP, SBP, and PC have been widely found to be biomarkers closely related to biological aging. Pinto [61] noted that elevated pulse pressure due to decreased DBP and increased SBP was the most potent risk predictor in older adults and was associated with older age. In epidemiological studies, aging populations were more likely to exhibit features of lower PC and higher platelet activity, which are associated with higher rates of cardiovascular disease [62–64]. The link between gender or height and aging was also frequently mentioned [65, 66]. In a study of conscripts from Italian inland villages, short people (height less than 161.1 cm) generally had higher survival rates than tall peers [67]. This may be related to caloric restriction, cell replication potential, telomere shortening, and cardiac pumping efficiency [67, 68]. What’s more, the gender-driven characteristics of aging have become the focus of current attention, with gender differences in life expectancy, biological aging, and frailty indices [69]. Of these, women are generally biologically younger than men, consistent with a lower BA assessed by molecular biomarkers [4].

Overall, the BA measurement model we developed integrated multidimensional biosignatures that more systematically reflected human aging. This line of evidence reinforces our findings and suggests that the variable screening results of the Stacking model are biologically interpretable. Besides, although fewer biological features are considered in the model, this facilitates the generalization and practical application of the model and its workflow.

The large sample data of Chinese medical examination data enables us to explore the influence of fitting on the stability of correlation results and develop a new composite BA prediction model after comparing the most suitable interpolation methods. Nonetheless, several limitations need to be discussed. First, although the interpolation methods explored in this study are convenient and practical, more novel missing value imputation methods can be further attempted to be transferred to the medical examination dataset [39], such as the variational AE applied to Genomic data imputation [38]. Second, our data lacked information on outcome variables (e.g., death) to establish a link between BA and survival analysis. We, therefore, associated BA with a health risk indicator that predicted mortality risk instead. Third, the training and test sets of the BA prediction model are both from the same dataset. Testing with external datasets will further evaluate the generalization ability of the ML-BAs [70]. Finally, the biological features used in the study were mostly limited to biochemical indicators, and aging-related indicators that have been discovered, such as mean corpuscular volume, are not included in our data. These may weaken the interpretability of predicted BA and fail to supplement the validation of more existing results [18, 71]. However, with the popularization of big medical data, phenotype information (e.g. cognitive level, gait speed [72, 73]), methylation data (e.g. CpG sites [74, 75]), metabolomic features and pathways (e.g. C-glycosyl tryptophan, α-ketoglutarate and TCA cycle [76–78]) will be more convenient, which assists in predicting and explaining the aging process more systematically. Therefore, as more dimensions of individual indicators are taken into account, our composite BA and its construction process will have a broader reference value.

## Conclusion

We found RRLR best suited for interpolation on our medical examination dataset, while AE exhibited the highest stability at high missing rates. We pointed out a potential problem of over-fitting affecting the association results in recently proposed ML-BAs. After comparing machine learning methods, we constructed two XGB-BAs with different fitting degrees on the training set (similar performance on the test set) to illustrate the degree of fit by the association between ML-BAs and health statuses that will affect the stability of BA application. For this case, we proposed a composite ML-BA based on the Stacking method with a simple meta-model (STK-BA), which overcame the overfitting problem, and associated more strongly with CA (r = 0.66, P < 0.001), healthy risk indicators, disease counts and six types of disease. Furthermore, we found that DBP, height, SBP, gender, and platelet content were the top five important biological features in STK-BA. However, the influence of the degree of overfitting on the longitudinal association results and the use of external data sets to test the generalization ability of STK-BA are lacking in our study, which deserves further exploration. Overall, our findings supported the application of ML in geriatric research and suggested improvements to existing ML-based BA models. This new aging measurement method captures aging characteristics beyond CA more stably, and provides new possibilities for future work such as the application of BA in risk stratification and aging intervention studies.

## Methods

### Study population and assessment of physical examination measurements

Based on the electronic health records of residents in Zhejiang Province, China, this study conducted a representative physical examination survey among different age groups. According to the national code for basic public health services, the records were established by substrate medical and health institutions, including township health centers and community health service centers, in 23 cities, and districts of Zhejiang Province.

This study selected potential age-related features missing under 80% and observations with features missing under 20%. Out of the 418,161 participants aged 30–100 years old, we excluded observations those included outliers in comparison with data of the same age and sex (N = 30,935) and those with more than 20% missing data on variates (N = 309,416), leaving the analytic sample of 77,810 adults. Middle age starts around age 45, while the very old are vulnerable to NCDs and socially disadvantaged [18, 79]. Additionally, due to the relatively small size of the oldest-old group and the differences between participants aged 45–90 and others, we excluded participants aged under 45 and over 90 (N = 666). A total of 77,144 participants with 17 biochemical indicators (i.e. systolic blood pressure (SBP), diastolic blood pressure (DBP), hemoglobin, white blood cell, platelets, fasting serum glucose (FSG), serum glutamic pyruvate transaminase (SGPT), serum glutamic oxaloacetic transaminase (SGOT), serum bilirubin, total cholesterol (TC), triglycerides (TG), total bilirubin, low-density lipoprotein (LDL), high-density lipoprotein (HDL), urine protein, urine sugar, urine ketone body, urine occult blood) and 5 physical indicators (i.e. gender, height, weight, waist, body mass index (BMI)) were included in the study. The above indicators were obtained from regular physical examinations. The biological features’ attributions of study populations were shown in Additional file 1: Table S14. The BMI was calculated as weight in kilograms divided by height in meters squared. The data of urine protein, urine sugar, urine ketone body, and urine occult blood were defined as positive and negative levels.

### Comparison of interpolation methods for missing values

Interpolating the missing values helps improve the model’s predictive power. Nevertheless, no specific interpolating method is universal. We compared the mean value, k-Nearest Neighbor (KNN), multiple imputations by chained equations (MICE), AutoEncoder (AE), and round-robin linear regression (RRLR) interpolation under the condition of missing completely at random MCAR) and missing not at random (MNAR) to choose the method that best fitted our data. Mean value, KNN, MICE, RRLE and AE respectively represent five typical interpolation methods: simple interpolation, unsupervised learning interpolation, multiple interpolation, regression interpolation, and deep learning network with generative ability methods [33, 38]. The different interpolation principles of these five methods make them applicable to different situations of missing medical data. Thus, it is of great significance to explore a more appropriate interpolation method for the specific missing data.

In real world medical examination data sets, the true values corresponding to the missing locations could not be obtained, nor could the accuracy of the filled value be intuitively evaluated. Therefore, in order to better evaluate the filling performance of different filling methods, we introduced missing values on real world data without missing values to carry out simulation experiments. The process of introducing and interpolating missing values is shown in Additional file 1: Fig. S3. The primary process is as follows:

(1) The missing ratio of each variable (variables with a missing ratio > 2% were considered) and the total missing ratio of all variables in the original data set were calculated, which were used to simulate the missing situation under MNAR and MCAR. (2) The samples without missing values (n = 37,320) were selected to form the simulation data set, of which 80% were used for training and adjusting core parameters of models (such as K in the KNN method), and 20% were used for testing and comparing results. The mean and variance of each variable in the training set were calculated. (3) Based on the results in (1), the missing ratio of different variables (MNAR) or random missing ratio (MCAR, 5%, 10%, 20%, 30%) were introduced into the simulation dataset, and the missing location information was recorded with matrices of the same size at the same time. (4) After interpolation, the imputed value of the test set of each method was compared with the true value by MSE and R^2^ (with a view to the dimensional difference between different variables, the results in (3) were used to standardize the variables). To reduce the influence of overfitting on the results, we further used tenfold cross-validation as a reliable criterion to evaluate the performance of different methods.

### Feature selection and BA calculation

To avoid the redundancy of latent features, lasso regression was used for feature selection first (the data was standardized to avoid dimensional effects). In the second step, Pearson’s correlation was applied to evaluate the correlation of each feature with CA, and features that did not show significant correlations with age (*P* > 0.05) were excluded.

Similar to that described in previous publications [18], A total of 19 selected biological features were used as independent variables to construct ML-BA. Our work considered machine learning methods (Multiple Linear Regression (MLR), Generalized Additive Models (GAM), Support Vector Machine (SVM), Adaboost, Gradient Boosting Decision Tree (GBDT), Light Gradient Boosting Machine (LGBM), Catboost, Xgboost, Extra Trees) and neural network methods (Deep Neural Networks (DNN), Convolutional Neural Network (CNN)) that can be used for regression analysis. The Pearson correlations, MAE, and RMSE between BA and CA are the indicators used to compare different BA estimation algorithms, which are done in the test set [25, 26].

Finally, stacking model fusion was performed using the top five performing models to calculate the final BA in years (base models). The meta-model considered MLR, GAM (spline regression), SVM, and random forest (RF). Meanwhile, the two xgboost-based BA was calculated, one took the parameters from the Stacking model (XGB-BA1); one amplifies the fit of the training set while keeping the test set results approximately unchanged (XGB-BA2). Both models were trained on the training set to predict the full data set and used to compare the effect of training set overfitting on BA.

**Fig. 6:**
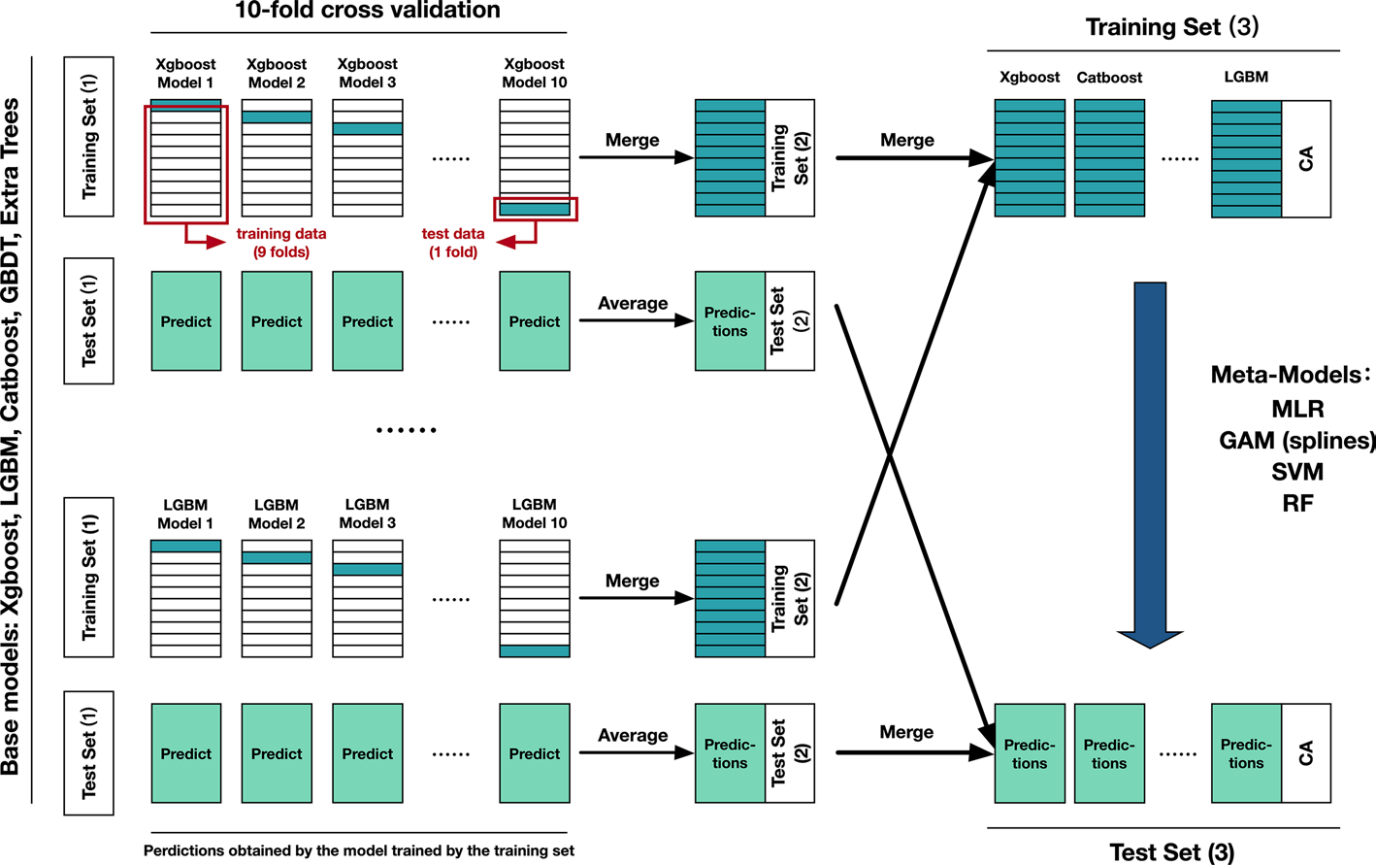
The schematic diagram of the Stacking method

A schematic diagram of the Stacking method was presented in Fig. 6. Specifically, the data was divided into the training set (1) and test set (1) with an 8:2 ratio, using CA as the response variable. Each base model was subjected to tenfold cross-validation in the training set (1), a total of 10 times (9 folds as training data for constructing models and a fold as test data per time). The merged result of the predicted values on each test data was the training set (2). The model obtained by each training data also predicted the test set (1), and the mean of the 10 results on the test set (1) was the test set (2). Both training set (2) and test set (2) were provided by the single base model. After repeating these steps for the selected five models, the combined training sets (2) and test sets (2) provided by different models were the training set (3) and test set (3) for the meta-model (The response variable was inherited from the initial training and test set). The training process of the meta-model was the same as that of the single model.

### The associations with general health statuses

The way to investigate the performance of estimated BAs in capturing health risk was to consider their possible relationship to known health risk indicators, or how estimated BAs differentiate between subjects with known disease and those without the disease.

Health risk indicators describe the general health state of an individual, such as the A Body Shape Index (ABSI) [46], Surface-based body shape index (SBSI) [80], waist-to-height ratio (WHtR), waist-to-hip ratio (WHR), etc. These indicators are associated with various mortality risks. Considering the biological features covered in the dataset, we used ABSI and WHtR as health risk indicators and further adjusted them for BMI, CA, and family disease. WHtR was obtained from the ratio of waist to height. ABSI was obtained by adjusting waist circumference (WC) for height and weight:

$${\text{ABSI}} = { }\frac{{{\text{WC}}}}{{{\text{BMI}}^{2/3} {\text{Height}}^{1/2} }}$$

For an effective BA model, when BA increases, the health risk indicator should show a corresponding upward trend. Rahman et al. [6] found a clear separation of BA acceleration by WHtR and SBSI categories (quartiles) in different BA predictive models.

Analyze whether BA will characterize any differences between healthy subjects and subjects with certain known chronic diseases [6, 28]. Individuals with more chronic diseases should have higher mean BA levels than people without any chronic diseases. There are 7 types of diseases diagnosed after physical examination, including cerebrovascular disease, kidney disease, heart disease, vascular disease, eye disease, nervous system disease, and other system diseases. We created a binary variable for each type of disease, with the disease marked as 1 and 0 otherwise. As described above, we added up the disease types of each individual to obtain a disease count variable (ranging from 0 to 7). After accounting for the population distribution, a three-category variable for disease counts was created, no disease, 1 disease, and 2 or more diseases.

### Statistical analysis

We trained and optimized BA using training data (80%) and compared the different model results with RMSE, R^2^, and MAE on test data (20%). All interpolation methods were implemented in Python. The Stacking method with the simple meta-model covering GBDT, LGBM, Catboost, Xgboost, and Extra Trees was selected to calculate the optimal BA (STK-BA) in years. To emphasize the advantages of the Stacking fusion model, the two Xgboost-based BAs (XGB-BAs) with different over-fitting in the training set were also introduced. Furthermore, to assess the importance of features to BA, the feature importance value (FIV) of the five models in the Stacking model was converted to weights and added together [18].

As shown in Fig. 1, we performed two primary analyses, one for health risk indicators, and one for disease counts and specific diseases. To account for confounding effects and to perform further subgroup analyses, we considered the following covariates: chronological age, family disease status, BMI. The details were provided in Additional file 1: Tables S14 and S15.

The associations between ML-BAs and health risk indicators were analyzed by MLR. And the health risk indicators were further classified according to quintiles (Q1–Q5) to compare whether the changes in BA are consistent with the increase of quantiles (Model 1 was a crude model, Model 2 was adjusted for CA, BMI, and family disease status).

To assess the associations between ML-BAs with full-sample disease counts, we first built the MLRs with ML-BAs as the dependent variable. Based on the results of the regression, we estimated BA increments for each disease count category compared with disease-free participants. Subsequently, we used Poisson regression models to examine the associations between ML-BAs and disease counts (the dependent variable). Moreover, the logistic regression model (with or without disease as the dependent variable) was used to assess the association of specific diseases with BAs. We considered two models: Model 1 was a crude model, Model 2 was adjusted for CA and family disease status.

For linear and Poisson regression models, we recorded coefficients, standard errors (SE), z-score, and P-values; for logistic regression models, we recorded odds ratios (ORs), corresponding 95% confidence intervals (95% CI), z-score, and P-values. Statistical analysis and visualization of all data were performed using R Version 4.1.2, Python Version 3.8.8, and Prism 8. Continuous variables were presented as mean ± SD, while categorical variables were presented as numbers (proportions). P < 0.05 (two-tailed) was considered statistically significant.

## Supplementary Information

**Additional file 1. Fig. S1**. Optimized lambda selection (A) and feature selection (B) in Lasso regression. **Fig. S2**. Feature importance values in the Stacking model. **Fig. S3**. Schematic diagrams of the process for introducing missing values and interpolation in MCAR (A) and MNAR (B). **Table S1**. Parameter optimization results of KNN and MICE in MCAR. **Table S2**. Parameter optimization results of KNN and MICE in MNAR. **Table S3**. The optimized parameters of AE and RRLR. **Table S4**. The interpolation time consumed by the different models. **Table S5**. Coefficients of biological features in Lasso regression (lambda = 0.00072). **Table S6**. Parameter optimization results of GAM (splines), AdaBoost, CNN, DNN, Extra Trees, GBDT, LGBM, CatBoost, XGBoosts. **Table S7**. The results and parameters of two XGBoost models. **Table S8**. The variable importance values of the sub-models in the Stacking model. **Table S9**. Associations of STK-BA and XGB-BAs with health risk indicators. **Table S10**. Associations of STK-BA and XGB-BAs with health risk indicators (Quintile, ABSI). **Table S11**. Associations of STK-BA and XGB-BAs with health risk indicators (Quintile, WHtR). **Table S12**. Predicted increase in STK-BA and XGB-BAs for each disease count. **Table S13**. The associations between each disease and STK-BA, XGB-BAs. **Table S14**. The 19 biological features’ attributes of study participants (n=77,144). Table S15. The disease status of study population (n=77, 144).

## Data Availability

The data that support the findings of this study are available from the Center for Disease Control of Zhejiang Province, but restrictions apply to the availability of these data, which were used under license for the current study, and so are not publicly available. However, data are available from the authors upon reasonable request and with permission of the Center for Disease Control of Zhejiang Province.

## Abbreviations

BA: Biological age
CA: Chronological age
ML-BA: Machine learning-based BA
STK-BA: BA based on Stacking method with a simple meta-model
XGB-BA: Xgboost-based BA
PCA: Principle component analysis
MLP: Multilayer perceptron
KDM: The Klemera and Doubal method
ML: Machine learning
SD: Standard deviation
XGBoost: Extreme gradient boosting
BMI: Body mass index
KNN: K-nearest neighbor
MICE: Multiple imputations by chained equations
AE: Autoencoder
RRLR: Round-robin linear regression
MCAR: Missing completely at random
MNAR: Missing not at random
MSE: Mean squared error
MLR: Multiple linear regression
GAM: Generalized additive models
SVM: Support vector machine
GBDT: Gradient boosting decision tree
LGBM: Light gradient boosting machine
DNN: Deep neural networks
CNN: Convolutional neural network
RMSE: Root mean squared error
MAE: Mean absolute error
DBP: Diastolic blood pressure
SBP: Systolic blood pressure
SGPT: Serum glutamic-pyruvic transaminase
SGOT: Serum glutamic-oxaloacetic transaminase
CI: Confidence interval
TC: Total cholesterol
TG: Triglycerides
LDL: Low-density lipoprotein
HDL: High density lipoprotein
PC: Platelets count
TCA cycle: Tricarboxylic acid cycle
ABSI: A Body Shape Index
SBSI: Surface-based body shape index
WHtR: Waist-to-height ratio
WHR: Waist-to-hip ratio
FIV: Feature importance value
ORs: Odds ratios
NCDs: Non-communicable chronic disease
LOOCV: Leave-one-out cross-validation
WC: Waist circumference
GCV: Generalized cross-validation
