# Supplementary material for: A machine learning-based data mining in medical examination data: a biological features-based biological age prediction model
Source: BMC Bioinformatics. 2022 Oct 3;23:411. doi: 10.1186/s12859-022-04966-7 (PMC9528174; doi:10.1186/s12859-022-04966-7)
Supplement: Supplementary file 1 — Additional file 1. Fig. S1. Optimized lambda selection (A) and feature selection (B) in Lasso regression. Fig. S2. Feature importance values in the Stacking model. Fig. S3. Schematic diagrams of the process for introducing missing values and interpolation in MCAR (A) and MNAR (B). Table S1. Parameter optimization results of KNN and MICE in MCAR. Table S2. Parameter optimization results of KNN and MICE in MNAR. Table S3. The optimized parameters of AE and RRLR. Table S4. The interpolation time consumed by the different models. Table S5. Coefficients of biological features in Lasso regression (lambda = 0.00072). Table S6. Parameter optimization results of GAM (splines), AdaBoost, CNN, DNN, Extra Trees, GBDT, LGBM, CatBoost, XGBoosts. Table S7. The results and parameters of two XGBoost models. Table S8. The variable importance values of the sub-models in the Stacking model. Table S9. Associations of STK-BA and XGB-BAs with health risk indicators. Table S10. Associations of STK-BA and XGB-BAs with health risk indicators (Quintile, ABSI). Table S11. Associations of STK-BA and XGB-BAs with health risk indicators (Quintile, WHtR). Table S12. Predicted increase in STK-BA and XGB-BAs for each disease count. Table S13. The associations between each disease and STK-BA, XGB-BAs. Table S14. The 19 biological features’ attributes of study participants (n=77,144). Table S15. The disease status of study population (n=77, 144). [file 12859_2022_4966_MOESM1_ESM.docx]

Supporting information for

**A machine learning-based data mining in medical examination data: a group features-based biological age prediction model**

**Qing Yang^1, †^, Sunan Gao^2, †^, Junfen Lin^1^, Ke Lyu^3^, Zexu Wu^3^, Yuhao Chen^3^, Yinwei Qiu^1^, Yanrong Zhao^1^, Wei Wang^1^, Tianxiang Lin^1^, Huiyun Pan^4^ and Ming Chen^3, 4,^ ***

**^1^** Zhejiang Provincial Center for Disease Control and Prevention, Hangzhou 310051, China

**^2^** College of Biosystems Engineering and Food Science, Zhejiang University, Hangzhou 310058, China

**^3^** College of Life Sciences, Zhejiang University, Hangzhou 310058, China

**^4^** The First Affiliated Hospital of School of Medicine, Zhejiang University, Hangzhou 310058, China

**^†^** These authors contributed equally to this work

**Fig. S1** Optimized lambda selection (A) and feature selection (B) in Lasso regression

**Fig. S2** Feature importance values in the Stacking model.

**Fig. S3** Schematic diagrams of the process for introducing missing values and interpolation in MCAR (A) and MNAR (B).

**Table S1.** Parameter optimization results of KNN and MICE in MCAR.

**Table S2.** Parameter optimization results of KNN and MICE in MNAR.

**Table S3.** The optimized parameters of AE and RRLR.

**Table S4.** The interpolation time consumed by the different models.

**Table S5.** Coefficients of biological features in Lasso regression (lambda = 0.00072).

**Table S6.** Parameter optimization results of GAM (splines), AdaBoost, CNN, DNN, Extra Trees, GBDT, LGBM, CatBoost, XGBoosts.

**Table S7.** The results and parameters of two XGBoost models.

**Table S8.** The variable importance values of the sub-models in the Stacking model.

**Table S9.** Associations of STK-BA and XGB-BAs with health risk indicators.

**Table S10.** Associations of STK-BA and XGB-BAs with health risk indicators (Quintile, ABSI).

**Table S11.** Associations of STK-BA and XGB-BAs with health risk indicators (Quintile, WHtR).

**Table S12.** Predicted increase in STK-BA and XGB-BAs for each disease count.

**Table S13.** The associations between each disease and STK-BA, XGB-BAs.

**Table S14.** The 19 biological features’ attributes of study participants (n=77,144).

**Table S15.** The disease status of study population (n=77, 144).

**
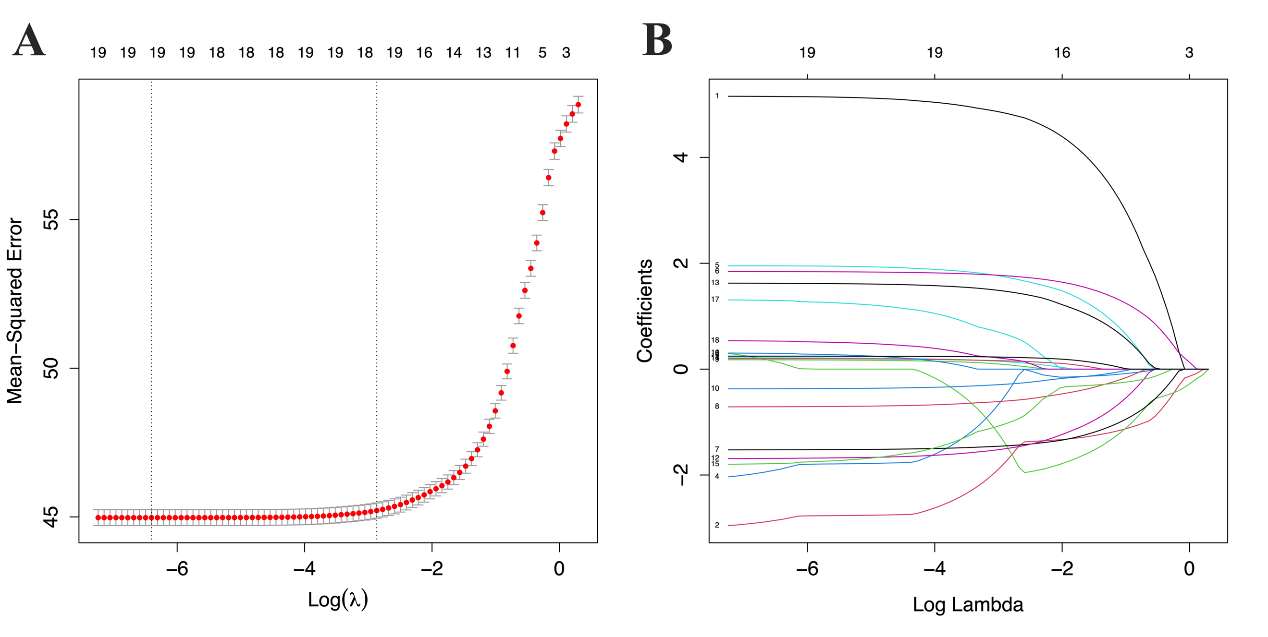
**

**Fig. S1** Optimized lambda selection (A) and feature selection (B) in Lasso regression.


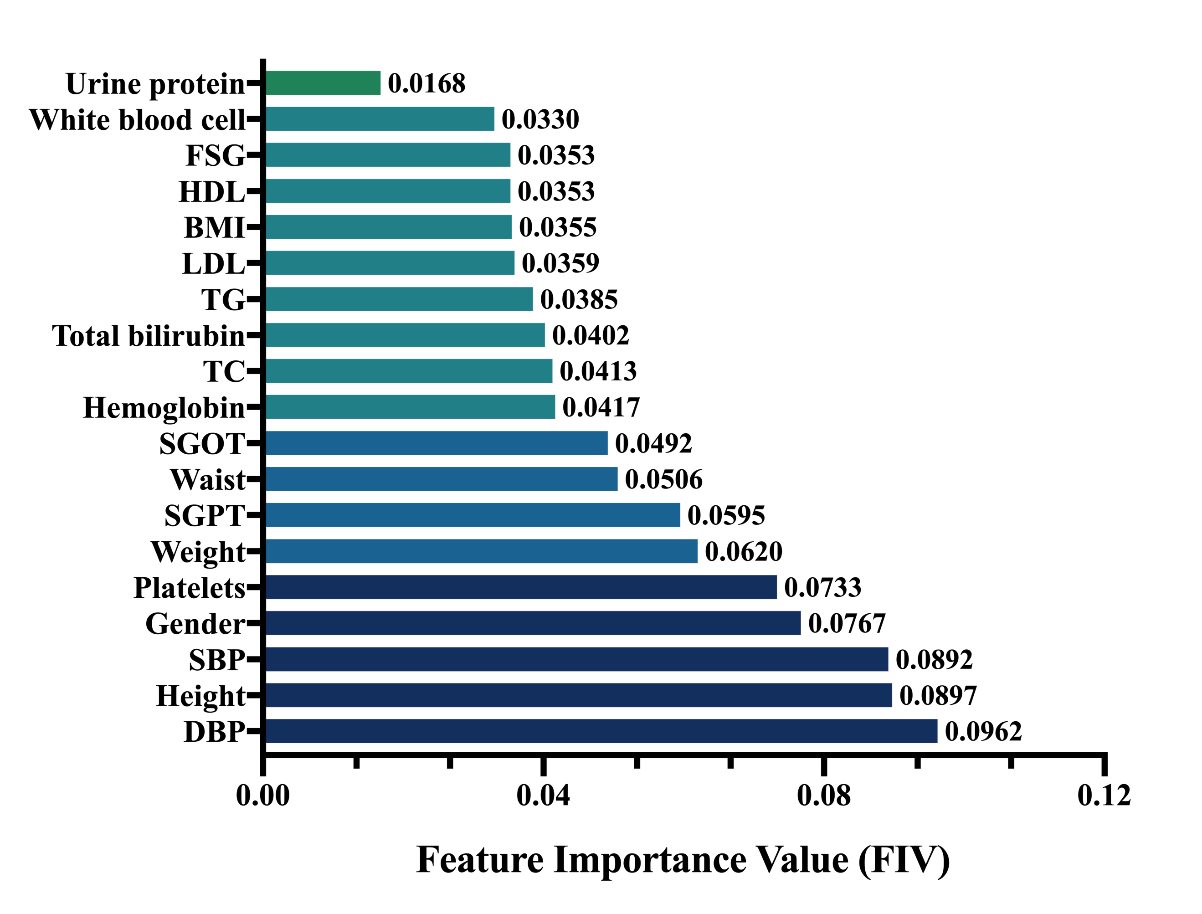


**Fig. S2** Feature importance values in the Stacking model.


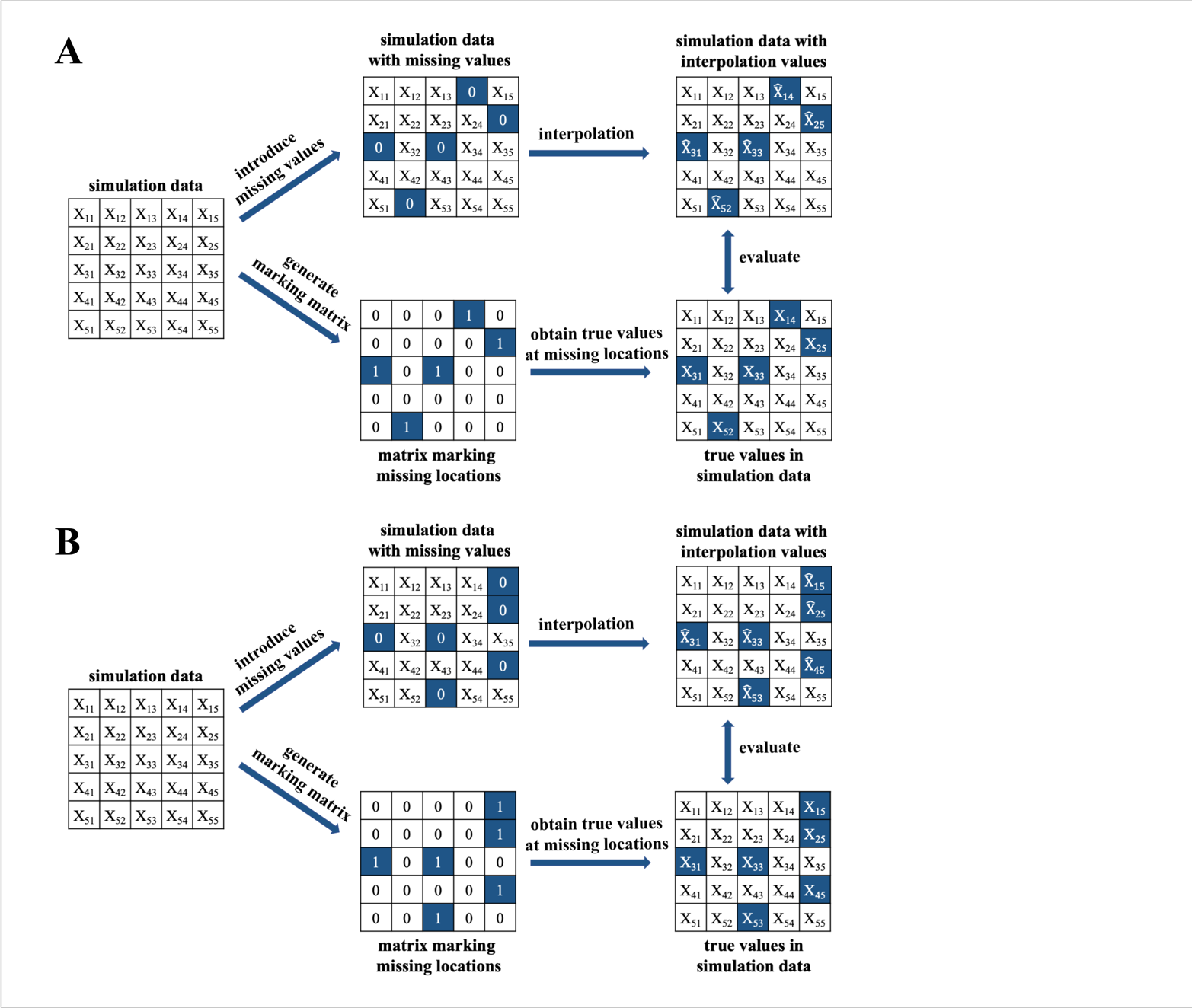
**Fig. S3** Schematic diagrams of the process for introducing missing values and interpolation in MCAR (A) and MNAR (B).

* In our clinical data, considering that only specific variables were missing more than 2%, indicating that missing data may only depend on the variables themselves, thus we performed MNAR simulation. In addition, considering that the study data were only a random subset of the study population and that the probability of missing data was not observed to be affected by other variables obviously, thus we performed MCAR simulations.

**Table S1.** Parameter optimization results of KNN and MICE in MCAR.

| Method | Missing Ratio | | Parameters | Train Set | | Valid Set | | |
| --- | --- | --- | --- | --- | --- | --- | --- | --- |
| KNN |  |  | | MSE | R^2^ | | MSE | R^2^ |
|  | 30% | 5 | | 1.1297 | 1.0312 | | -0.1291 | -0.0117 |
|  |  | 15 | | 1.1148 | 1.0233 | | -0.1097 | -0.0229 |
|  |  | 30 | | 1.1393 | 1.0306 | | -0.1347 | -0.0377 |
|  |  | 50 | | 1.1224 | 1.0294 | | -0.1222 | -0.0222 |
|  | 20% | 5 | | 1.0405 | 0.9458 | | -0.0325 | 0.0505 |
|  |  | 15 | | 1.0408 | 0.9302 | | -0.0541 | 0.0617 |
|  |  | 30 | | 1.0312 | 0.9254 | | -0.0234 | 0.0661 |
|  |  | 50 | | 1.0458 | 0.9509 | | -0.0424 | 0.0445 |
|  | 10% | 5 | | 0.9705 | 0.9015 | | 0.043 | 0.0865 |
|  |  | 15 | | 0.9369 | 0.8869 | | 0.062 | 0.0964 |
|  |  | 30 | | 0.963 | 0.8605 | | 0.042 | 0.1307 |
|  |  | 50 | | 0.9549 | 0.8751 | | 0.0347 | 0.0894 |
|  | 5% | 5 | | 0.9288 | 0.8771 | | 0.0696 | 0.1439 |
|  |  | 15 | | 0.9366 | 0.8427 | | 0.093 | 0.1112 |
|  |  | 30 | | 0.9124 | 0.8336 | | 0.0666 | 0.1724 |
|  |  | 50 | | 0.9146 | 0.8741 | | 0.094 | 0.135 |
| MICE | 30% | 1 | | 1.0346 | 1.0331 | | -0.0303 | -0.0401 |
|  |  | 3 | | 0.9143 | 0.9113 | | 0.0856 | 0.0868 |
|  |  | 5 | | 0.8837 | 0.8806 | | 0.1119 | 0.1156 |
|  |  | 10 | | 0.8797 | 0.865 | | 0.1232 | 0.124 |
|  | 20% | 1 | | 0.9652 | 0.9738 | | 0.0328 | 0.0399 |
|  |  | 3 | | 0.8726 | 0.8582 | | 0.1349 | 0.1293 |
|  |  | 5 | | 0.8375 | 0.8512 | | 0.1613 | 0.1531 |
|  |  | 10 | | 0.8369 | 0.8403 | | 0.1664 | 0.152 |
|  | 10% | 1 | | 0.8901 | 0.9017 | | 0.1088 | 0.0792 |
|  |  | 3 | | 0.8168 | 0.8071 | | 0.1886 | 0.186 |
|  |  | 5 | | 0.7825 | 0.8164 | | 0.2065 | 0.1999 |
|  |  | 10 | | 0.7956 | 0.809 | | 0.2139 | 0.198 |
|  | 5% | 1 | | 0.8644 | 0.8907 | | 0.1341 | 0.1305 |
|  |  | 3 | | 0.8061 | 0.7553 | | 0.2193 | 0.2034 |
|  |  | 5 | | 0.7445 | 0.7804 | | 0.2384 | 0.2253 |
|  |  | 10 | | 0.7668 | 0.7852 | | 0.2404 | 0.223 |

* The nearest neighbor value for KNN was chosen from 5, 15, 30, and 50, and the number of mean match candidates for MICE was chosen from 1, 3, 5, and 10.

**Table S2.** Parameter optimization results of KNN and Mice in MNAR.

| Method | Parameters | Train Set | | Valid Set | |
| --- | --- | --- | --- | --- | --- |
| KNN |  | MSE | R^2^ | MSE | R^2^ |
|  | 5 | 1.0195 | 1.0191 | -0.0223 | -0.025 |
|  | 15 | 0.9149 | 0.9057 | 0.0826 | 0.0891 |
|  | 30 | 0.8912 | 0.8853 | 0.1063 | 0.1096 |
|  | 50 | 0.8848 | 0.8866 | 0.1128 | 0.1083 |
| MICE | 1 | 0.9253 | 0.8396 | 0.0721 | 0.1555 |
|  | 3 | 0.9189 | 0.8377 | 0.0786 | 0.1574 |
|  | 5 | 0.9173 | 0.8064 | 0.0801 | 0.1889 |
|  | 10 | 0.9273 | 0.8426 | 0.0701 | 0.1525 |

* The nearest neighbor value for KNN was chosen from 5, 15, 30, and 50, and the number of mean match candidates for MICE was chosen from 1, 3, 5, and 10.

**Table S3.** The optimized parameters of AE and RRLR.

|  | Optimized parameters |
| --- | --- |
| RRLR | max_iter = 20, tol = 0.001, n_nearest_features = None, initial_strategy = 'median',  imputation_order = 'ascending' (IterativeImputer function in Python) |
| AE (MCAR) | batch size = 64, epochs = 200, Encoder = {64,16}, Decoder = {23},  optimizer = adam, loss = mse, metrics = mae, learning rate = 0.001,  activation = relu (keras package in Python) |
| AE (MNAR) | batch size = 32, epochs = 150, Encoder = {64,16}, Decoder = {23},  optimizer = adam, loss = mse, metrics = mae, learning rate = 0.001,  activation = relu (keras package in Python) |

**Table S4.** The interpolation time consumed by the different models.

|  | Missing ratio | | Time consumption (s) | | | | |
| --- | --- | --- | --- | --- | --- | --- | --- |
| MCAR |  | Mean | | KNN | RRLR | MICE | AE |
|  | 30% | 4.2 | | 15.2 | 8.4 | 43 | 1.7 |
|  | 20% | 7.3 | | 9.6 | 9.4 | 54 | 1.3 |
|  | 10% | 5.9 | | 5.1 | 6.1 | 56 | 1.1 |
|  | 5% | 2.7 | | 4.2 | 11.5 | 10 | 1.2 |
| MNAR | / | 1.7 | | 4.6 | 2.1 | 17.9 | 2.4 |

* The time it took to interpolate once based on optimized parameters on Macbook pro (3.1 GHz Intel Core i5, 8 GB 2133 MHz).

**Table S5.** Coefficients of biological features in Lasso regression (lambda = 0.00166).

| Variable | Coefficient | Variable | Coefficient |
| --- | --- | --- | --- |
| Gender | 5.150 | SGPT | -1.685 |
| Height | -2.839 | SGOT | 1.622 |
| Weight | 0.111 | Total bilirubin | 0.197 |
| BMI | -1.884 | Total cholesterol | -1.781 |
| Waist | 1.951 | Triglyceride | 0.296 |
| SBP | 1.842 | LDL | 1.295 |
| DBP | -1.524 | HDL | 0.529 |
| Hemoglobin | -0.710 | Urine protein | 0.245 |
| White blood cell | 0.175 | Urine sugar | . |
| Platelet | -0.370 | Urine ketone bodies | . |
| Fasting serum glucose | 0.213 | Urine occult blood | . |

**Table S6.** Parameter optimization results of GAM (splines), AdaBoost, CNN, DNN, Extra Trees, GBDT, LGBM, CatBoost, XGBoosts.

|  | Optimized parameters |
| --- | --- |
| GAM(splines) | Smooth spline regression with 4 degrees of freedom was adopted for all variables |
| AdaBoost | n_estimators=1000, learning_rate=0.29, loss = ‘linear’ |
| CNN | batch size = 64, epochs = 250, optimizer = adam, loss = mse, activation = relu  Layer = {Conv1D(128,3), Maxpooling(2), Conv1D(64, 3), MaxPooling1D(2), layers.Flatten(), Dense(32), Dense(1)} (keras package in Python) |
| DNN | batch size = 50, epochs = 100, loss = mse, activation = relu  optimizer = adam_v2.Adam(learning_rate=0.0001, decay=lr/epochs)  Layer = {64, 32, 32, 1} (keras package in Python) |
| Extra Trees | n_estimators=100, criterion="mse", min_samples_split=2, min_samples_leaf=1 |
| GBDT | n_estimators=1500, loss='ls', learning_rate=0.1, subsample=1, max_depth=3  criterion='friedman_mse', min_samples_split=2, min_samples_leaf=1 |
| LGBM | n_estimators=1500, boosting_type='gbdt', num_leaves=31, max_depth=6  learning_rate=0.1, min_child_weight=0.001, min_child_samples=20, subsample=1 |
| CatBoost | Iterations=3000, border_count=235, depth=9, l2_leaf_reg=10.66  learn_rate=0.077, loss_function='RMSE' |
| Xgboost | n_estimators=2000, learning_rate=0.2, max_depth=3, min_child_weight=5 |

*Parameters that are not displayed are the default parameters in the model package.

**Table S7.** The results and parameters of two XGBoost models.

|  | Training set | | | | Test set | | | |
| --- | --- | --- | --- | --- | --- | --- | --- | --- |
|  | RMSE | R^2^ | MAE | Pearson  correlation | RMSE | R^2^ | MAE | Pearson  correlation |
| XGB-BA1 | 4.988 | 0.578 | 3.780 | 0.760 | 5.869 | 0.414 | 4.489 | 0.643 |
| XGB-BA2 | 3.915 | 0.740 | 2.890 | 0.860 | 5.918 | 0.404 | 4.507 | 0.636 |
|  | Model parameter | | | | | | | |
| XGB-BA1 | n_estimators=2000, learning_rate=0.2, max_depth=3, min_child_weight=5 | | | | | | | |
| XGB-BA2 | n_estimators=2500, learning_rate=0.2, max_depth=4, min_child_weight=5 | | | | | | | |

*Parameters that are not displayed are the default parameters in the model package.

**Table S8.** The variable importance values of the sub-models in the Stacking model.

| Feature | Xgboost | GBDT | ETR | LGBM | Catboost | Total |
| --- | --- | --- | --- | --- | --- | --- |
| DBP | 0.0725 | 0.1314 | 0.0844 | 0.0782 | 0.1144 | 0.0962 |
| Height | 0.0870 | 0.1291 | 0.1071 | 0.0574 | 0.0679 | 0.0897 |
| SBP | 0.0788 | 0.1211 | 0.0749 | 0.0658 | 0.1056 | 0.0892 |
| Gender | 0.2206 | 0.0799 | 0.0220 | 0.0098 | 0.0514 | 0.0767 |
| Platelets | 0.0660 | 0.0949 | 0.0746 | 0.0657 | 0.0655 | 0.0733 |
| Weight | 0.0633 | 0.0769 | 0.0699 | 0.0514 | 0.0484 | 0.0620 |
| SGPT | 0.0632 | 0.0739 | 0.0516 | 0.0504 | 0.0584 | 0.0595 |
| Waistline | 0.0538 | 0.0448 | 0.0589 | 0.0429 | 0.0527 | 0.0506 |
| SGOT | 0.0481 | 0.0495 | 0.0487 | 0.0484 | 0.0513 | 0.0492 |
| hemoglobin | 0.0270 | 0.0254 | 0.0545 | 0.0569 | 0.0447 | 0.0417 |
| TC | 0.0229 | 0.0334 | 0.0419 | 0.0613 | 0.0469 | 0.0413 |
| Total bilirubin | 0.0220 | 0.0222 | 0.0441 | 0.0656 | 0.0472 | 0.0402 |
| TG | 0.0208 | 0.0252 | 0.0429 | 0.0599 | 0.0435 | 0.0385 |
| LDL | 0.0172 | 0.0183 | 0.0399 | 0.0605 | 0.0438 | 0.0359 |
| BMI | 0.0210 | 0.0223 | 0.0440 | 0.0540 | 0.0361 | 0.0355 |
| HDL | 0.0148 | 0.0166 | 0.0416 | 0.0618 | 0.0417 | 0.0353 |
| FSG | 0.0194 | 0.0162 | 0.0438 | 0.0563 | 0.0406 | 0.0353 |
| White blood cells | 0.0197 | 0.0151 | 0.0424 | 0.0520 | 0.0360 | 0.0330 |
| Urine protein | 0.0618 | 0.0038 | 0.0128 | 0.0018 | 0.0038 | 0.0168 |

**Table S9.** Associations of STK-BA and XGB-BAs with health risk indicators.

|  |  | Model 1 | | | Model 2 | | |
| --- | --- | --- | --- | --- | --- | --- | --- |
|  |  | Coef (SE) | t-value | P | Coef (SE) | t-value | P |
| ABSI | STK-BA | 30.85 (0.32) | 97.23 | <0.001 | 17.62 (0.25) | 69.94 | <0.001 |
|  | XGB1-BA | 30.27 (0.33) | 92.63 | <0.001 | 15.09 (0.23) | 64.82 | <0.001 |
|  | XGB2-BA | 30.46 (0.36) | 83.99 | <0.001 | 11.87 (0.22) | 54.50 | <0.001 |
| WHtR | STK-BA | 14.88 (0.33) | 45.53 | <0.001 | 30.31 (0.35) | 86.80 | <0.001 |
|  | XGB1-BA | 14.53 (0.34) | 43.34 | <0.001 | 25.96 (0.32) | 80.36 | <0.001 |
|  | XGB2-BA | 14.58 (0.37) | 39.46 | <0.001 | 20.27 (0.30) | 66.77 | <0.001 |

**Table S10.** Associations of STK-BA and XGB-BAs with health risk indicators (Quintile, ABSI).

| Age | Model | ABSI (Quintile) | | | | |  |  |  |  |
| --- | --- | --- | --- | --- | --- | --- | --- | --- | --- | --- |
|  |  | Q1 | Q2 | | Q3 | | Q4 | | Q5 | |
|  |  |  | Coef (SE) | P | Coef (SE) | P |  |  |  |  |
| STK-BA | Model 1 | Ref | 0.765 (0.054) | <0.001 | 1.467 (0.054) | <0.001 | 2.500 (0.054) | <0.001 | 4.693 (0.054) | <0.001 |
|  | Model 2 |  | 0.436 (0.041) | <0.001 | 0.850 (0.041) | <0.001 | 1.462 (0.042) | <0.001 | 2.674 (0.043) | <0.001 |
| XGB-BA1 | Model 1 | Ref | 0.760 (0.056) | <0.001 | 1.460 (0.056) | <0.001 | 2.499 (0.056) | <0.001 | 4.623 (0.056) | <0.001 |
|  | Model 2 |  | 0.386 (0.038) | <0.001 | 0.747 (0.038) | <0.001 | 1.300 (0.038) | <0.001 | 2.308 (0.039) | <0.001 |
| XGB-BA2 | Model 1 | Ref | 0.729 (0.062) | <0.001 | 1.466 (0.062) | <0.001 | 2.509 (0.062) | <0.001 | 4.647 (0.062) | <0.001 |
|  | Model 2 |  | 0.271 (0.036) | <0.001 | 0.589 (0.036) | <0.001 | 1.028 (0.036) | <0.001 | 1.812 (0.037) | <0.001 |

**Table S11.** Associations of STK-BA and XGB-BAs with health risk indicators (Quintile, WHtR).

| Age | Model | WHtR (Quintile) | | | | |  |  |  |  |
| --- | --- | --- | --- | --- | --- | --- | --- | --- | --- | --- |
|  |  | Q1 | Q2 | | Q3 | | Q4 | | Q5 | |
|  |  |  | Coef (SE) | P | Coef (SE) | P | Coef (SE) | P | Coef (SE) | P |
| STK-BA | Model 1 | Ref | 0.233 (0.057) | <0.001 | 0.548 (0.057) | <0.001 | 0.921 (0.057) | <0.001 | 2.183 (0.057) | <0.001 |
|  | Model 2 |  | 0.867 (0.042) | <0.001 | 1.514 (0.044) | <0.001 | 2.262 (0.047) | <0.001 | 4.035 (0.046) | <0.001 |
| XGB-BA1 | Model 1 | Ref | 0.207 (0.058) | <0.001 | 0.528 (0.058) | <0.001 | 0.880 (0.058) | <0.001 | 2.134 (0.058) | <0.001 |
|  | Model 2 |  | 0.720 (0.039) | <0.001 | 1.293 (0.041) | <0.001 | 1.921 (0.044) | <0.001 | 3.465 (0.051) | <0.001 |
| XGB-BA2 | Model 1 | Ref | 0.266 (0.064) | <0.001 | 0.545 (0.064) | <0.001 | 0.867 (0.064) | <0.001 | 2.163 (0.064) | <0.001 |
|  | Model 2 |  | 0.609 (0.037) | <0.001 | 1.023 (0.038) | <0.001 | 1.476 (0.041) | <0.001 | 2.734 (0.048) | <0.001 |

**Table S12**. Predicted increase in STK-BA and XGB-BAs for each disease count.

| Age | Model | Disease Counts | | | | |
| --- | --- | --- | --- | --- | --- | --- |
|  |  | 0 | 1 | | 2+ | |
|  |  |  | Coef (SE) | P | Coef (SE) | P |
| STK-BA | Model 1 | Reference | 0.998 (0.036) | <0.001 | 2.422 (0.136) | <0.001 |
|  | Model 2 |  | 0.170 (0.028) | <0.001 | 0.461 (0.103) | <0.001 |
| XGB-BA1 | Model 1 | Reference | 1.053 (0.038) | <0.001 | 2.623 (0.140) | <0.001 |
|  | Model 2 |  | 0.100 (0.025) | <0.001 | 0.372 (0.095) | <0.001 |
| XGB-BA2 | Model 1 | Reference | 1.240 (0.041) | <0.001 | 3.047 (0.153) | <0.001 |
|  | Model 2 |  | 0.069 (0.024) | 0.004 | 0.284 (0.088) | 0.001 |

**Table S13.** The associations between each disease and STK-BA, XGB-BAs

|  | Model 1 | | | Model 2 | | |
| --- | --- | --- | --- | --- | --- | --- |
|  | OR (95% CI) | z-score | P | OR (95% CI) | z-score | P |
| Kidney |  |  |  |  |  |  |
| STK-BA | 1.05 (1.01, 1.10) | 2.23 | 0.026 | 1.07 (1.00, 1.13) | 2.11 | 0.035 |
| XGB1-BA | 1.04 (1.00, 1.09) | 1.86 | 0.063 | 1.05 (0.99, 1.13) | 1.68 | 0.092 |
| XGB2-BA | 1.03 (0.99, 1.07) | 1.00 | 0.143 | 1.04 (0.97, 1.12) | 1.16 | 0.247 |
| Vascular |  |  |  |  |  |  |
| STK-BA | 1.02 (0.99, 1,04) | 1.28 | 0.202 | 0.98 (0.95, 1.01) | -1.30 | 0.190 |
| XGB1-BA | 1.01 (0.99, 1.04) | 1.23 | 0.209 | 0.97 (0.94, 1.00) | -1.89 | 0.059 |
| XGB2-BA | 1.02 (1.00, 1.04) | 1.52 | 0.128 | 0.96 (0.93, 0.99) | -2.30 | 0.021 |
| Heart |  |  |  |  |  |  |
| STK-BA | 1.09 (1.07, 1.11) | 10.14 | <0.001 | 1.02 (1.00, 1.05) | 2.18 | 0.030 |
| XGB1-BA | 1.10 (1.08, 1.11) | 11.88 | <0.001 | 1.04 (1.01, 1.06) | 3.00 | 0.002 |
| XGB2-BA | 1.09 (1.08, 1.11) | 12.51 | <0.001 | 1.04 (1.01, 1.06) | 2.72 | 0.007 |
| Cerebrovascular |  |  |  |  |  |  |
| STK-BA | 1.10 (1.09, 1.12) | 15.43 | <0.001 | 1.05 (1.03, 1.06) | 5.23 | <0.001 |
| XGB1-BA | 1.10 (1.09, 1.12) | 16.33 | <0.001 | 1.04 (1.02, 1.06) | 4.61 | <0.001 |
| XGB2-BA | 1.10 (1.09, 1.11) | 17.10 | <0.001 | 1.04 (1.02, 1.06) | 4.21 | <0.001 |
| Eye |  |  |  |  |  |  |
| STK-BA | 1.14 (1.10, 1.18) | 7.61 | <0.001 | 1.09 (1.04, 1.14) | 3.84 | <0.001 |
| XGB1-BA | 1.12 (1.09, 1.16) | 7.33 | <0.001 | 1.07 (1.02, 1.12) | 2.85 | 0.004 |
| XGB2-BA | 1.11 (1.08, 1.14) | 7.34 | <0.001 | 1.07 (1.01, 1.12) | 1.88 | 0.060 |
| Nervous System |  |  |  |  |  |  |
| STK-BA | 0.93 (0.89, 0.97) | -3.22 | 0.001 | 1.08 (1.02, 1.15) | 2.77 | 0.006 |
| XGB1-BA | 0.90 (0.87, 0.94) | -4.76 | <0.001 | 1.06 (1.00, 1.13) | 1.87 | 0.062 |
| XGB2-BA | 0.89 (0.86, 0.93) | -5.77 | <0.001 | 1.05 (0.99, 1.12) | 1.62 | 0.105 |
| Other System |  |  |  |  |  |  |
| STK-BA | 1.04 (1.03, 1.04) | 24.77 | <0.001 | 1.01 (1.01, 1.01) | 5.14 | <0.001 |
| XGB1-BA | 1.04 (1.03, 1.04) | 25.47 | <0.001 | 1.01 (1.00, 1.01) | 3.00 | 0.003 |
| XGB2-BA | 1.04 (1.03, 1.04) | 27.39 | <0.001 | 1.00 (1.00, 1.01) | 2.02 | 0.044 |

**Table S14.** The 19 biological features’ attributes of study participants (n=77,144).

| Variables | <60  years old | 60-70  years old | 70-80  years old | >80  years old | P |
| --- | --- | --- | --- | --- | --- |
| Males, n(%) | 2830  (26.1) | 20240  (49.3) | 9627  (48.3) | 2223  (42.0) | <0.001 |
| ***Body measurement*** |  |  |  |  |  |
| Height (cm) | 159  (155,164) | 160  (154,165) | 158  (151,164) | 155  (149,162) | <0.001 |
| Weight (kg) | 60.0  (54.7,67.0) | 60.2  (54.8,67.1) | 58.0  (52.0,65.0) | 54.7  (48.0,61.4) | <0.001 |
| BMI (kg/m2) | 23.7  (21.8,25.8) | 23.7  (21.8,25.8) | 23.4  (21.4,25.6) | 22.6  (20.6,24.7) | <0.001 |
| Waist (cm) | 81  (76,86) | 82  (77,88) | 82  (77,88) | 81  (76,87) | <0.001 |
| SBP (mmHg) | 133  (125,140) | 136  (128，145) | 139  (130,148) | 140  (130,150) | <0.001 |
| DBP (mmHg) | 82  (77,87) | 81  (77,86) | 80  (75,85) | 79  (74,84) | <0.001 |
| ***Blood routine examination*** | | | | | |
| Hemoglobin (g/L) | 135  (127,144) | 137  (128,148) | 135  (125,145) | 130  (121,140) | <0.001 |
| White blood cell (×10^9/L) | 5.5  (4.7,6.5) | 5.5  (4.7,6.6) | 5.5  (4.6,6.5) | 5.5  (4.6,6.5) | <0.001 |
| Platelets (×10^9/L) | 188  (150,226) | 179  (145,215) | 171  (136,208) | 162  (129,201) | <0.001 |
| ***Serum biochemical indexes*** | | | | | |
| Triglycerides (mmol/L) | 1.32  (1.00,1.90) | 1.23  (0.90,1.75) | 1.20  (0.90,1.63) | 1.12  (0.90,1.54) | <0.001 |
| Total bilirubin (mmol/L) | 13.1  (9.7,17.5) | 13.8  (10.3,18.2) | 14.1  (10.6,18.5) | 13.8  (10.2,18.0) | <0.001 |
| Total cholesterol (mmol/L) | 5.06  (4.48,5.6) | 4.94  (4.34,5.58) | 4.84  (4.23,5.49) | 4.74  (4.14,5.39) | <0.001 |
| Urine protein, n(%) | 538  (5.0) | 2864  (7.0) | 1846  (9.3) | 525  (9.9) | <0.001 |
| HDL (mmol/L) | 1.40  (1.21,1.59) | 1.41  (1.22,1.61) | 1.42  (1.22,1.63) | 1.44  (1.22,1.65) | <0.001 |
| LDL (mmol/L) | 2.79  (2.35,3.28) | 2.77  (2.32,3.25) | 2.72  (2.26,3.23) | 2.68  (2.22,3.19) | <0.001 |
| FSG (mmol/L) | 5.2  (4.8,5.7) | 5.2  (4.8,5.8) | 5.2  (4.8,5.8) | 5.2  (4.7,5.8) | <0.001 |
| SGPT (U/L) | 18  (14,24) | 18  (14,24) | 17  (13,23) | 15  (12,21) | <0.001 |
| SGOT (U/L) | 21  (17,25) | 22  (19,26) | 23  (19,28) | 23  (19,28) | <0.001 |

a. Urine protein was defined into two levels, positive and negative.

b. Compared by using Kruskal-Wallis tests (for continuous variables) and Chi-square tests (for categorical variables).

**Table S15.** The disease status of study population (n=77, 144).

| Variables | <60 | 60-70 | 70-80 | >80 | P |
| --- | --- | --- | --- | --- | --- |
|  | years old | years old | years old | years old |  |
| Family disease, n(%) | 1143(10.1) | 4185(10.2) | 2018(10.1) | 499(9.4) | <0.001 |
| Disease count, n(%) |  |  |  |  | <0.001 |
| 0 | 7066(65.1) | 23907(58.2) | 9245(46.4) | 2468(46.6) |  |
| 1 | 3713(34.2) | 16581(40.4) | 10130(50.8) | 2642(50.0) |  |
| >=2 | 77(0.71) | 573(1.40) | 555(2.78) | 187(3.53) |  |
| ***Specific disease*** |  |  |  |  |  |
| Cerebrovascular disease, n(%) | 58(0.53) | 437(1.06) | 467(2.34) | 174(3.28) | <0.001 |
| Kidney disease, n(%) | 32(0.29) | 128(0.31) | 96(0.48) | 39(0.74) | <0.001 |
| Heart disease, n(%) | 45(0.41) | 296(0.72) | 264(1.32) | 118(2.23) | <0.001 |
| Vascular disease, n(%) | 53(0.49) | 251(0.61) | 163(0.82) | 61(1.15) | <0.001 |
| Eye disease, n(%) | 30(0.28) | 138(0.34) | 137(0.69) | 50(0.94) | <0.001 |
| Nervous system disease, n(%) | 30(0.28) | 35(0.09) | 6(0.03) | 1(0.02) | <0.001 |
| Other system diseases, n(%) | 3745(34.5) | 16918(41.2) | 10498(52.7) | 2758(52.1) | <0.001 |

Disease counts were defined into three levels, “0”, “1”, and “>=2”. The level “>=2” includes disease counts equal to 2,3, and 4.

1. Compared by using Chi-square tests (for categorical variables).
